# Host-microbiota matching and epigenetic modulation drive *Daphnia magna* responses to cyanobacterial stress

**DOI:** 10.1093/ismejo/wraf247

**Published:** 2025-11-03

**Authors:** Karen Bisschop, Naina Goel, Manon Coone, Isabel Vanoverberghe, Anna Greffe, Jana Asselman, Ellen Decaestecker

**Affiliations:** Department of Biology, MicrobiomeEcoEvo group, IRF Life Sciences, KU Leuven KULAK, Kortrijk, WVL 8500, Belgium; Department of Biology, Ghent University, Ghent, OVL 9000, Belgium; Department of Biology, MicrobiomeEcoEvo group, IRF Life Sciences, KU Leuven KULAK, Kortrijk, WVL 8500, Belgium; Blue Growth Research Lab, Ghent University, Ostend Science Park, Ostend, WVL 8400, Belgium; Department of Biology, MicrobiomeEcoEvo group, IRF Life Sciences, KU Leuven KULAK, Kortrijk, WVL 8500, Belgium; Department of Biology, MicrobiomeEcoEvo group, IRF Life Sciences, KU Leuven KULAK, Kortrijk, WVL 8500, Belgium; Department of Microbiology, Immunology and Transplantation, Laboratory of Molecular Bacteriology (Rega Institute), KU Leuven, Leuven, VBR 3000, Belgium; Blue Growth Research Lab, Ghent University, Ostend Science Park, Ostend, WVL 8400, Belgium; Department of Biology, MicrobiomeEcoEvo group, IRF Life Sciences, KU Leuven KULAK, Kortrijk, WVL 8500, Belgium

**Keywords:** reciprocal transplant, DNA methylation, stress adaptation, life-history traits, microbial community structure, metagenomic profiling, aquatic microbiology, epigenetic plasticity, microbiota-reducing treatment, dysbiosis

## Abstract

Microbial communities are crucial in host adaptation to stressors, particularly in dynamic ecosystems. In aquatic environments, *Daphnia magna* is ideal for studying host-microbiome interactions due to its ecological importance and sensitivity. Adaptation to toxins, such as those produced by cyanobacteria, may involve both host and microbial gene repertoires. Yet, the influence of microbiota composition and function on host performance remains poorly understood. Because epigenetic mechanisms such as DNA methylation regulate gene expression and mediate adaptive responses, we also investigated whether these associations are reflected in DNA methylation levels. To address this, we conducted a fully factorial transplant experiment using microbiota-depleted *Daphnia* colonised with microbiota from the same or different genotype, previously exposed to toxic or nontoxic diets, or left uncolonised. We assessed life-history traits, microbial composition (16S rRNA genes), functional profiles (whole-genome-resequencing), and DNA methylation (colorimetric quantification). *Daphnia* fed nontoxic diets grew larger and reproduced more. Increased methylation occurred when microbiota donors differed from the host genotype and was strongest under toxic diet. Dysbiosis and reduced performance were noted in individuals colonised with toxic-diet microbiota from another genotype, where *Limnohabitans* spp. was reduced or absent. Signs of hormesis emerged when *Daphnia* received microbiota from their own genotype reared on nontoxic diets. DNA methylation of both host and microbiota was associated with functional pathways, including increased mitochondrial fatty acid biosynthesis. These findings highlight the importance of host-microbiota matching and microbial environmental history in shaping host performance and epigenetic responses, emphasizing the need to consider host-microbe-environment interactions in evolutionary and ecological studies.

## Introduction

Host-microbiome interactions are central to host performance and adaptation, especially in changing environments [1, 2]. Environmentally acquired microbiota [3] provide a flexible, nongenetic mechanism for rapid adaptation, underpinning concepts like the “extended genotype”, where symbionts influence host phenotype and plasticity [4]. Adaptation can thus arise from shifts in microbial communities rather than host genetic change [5, 6], with specific host-microbe combinations enhancing performance and persisting across generations [7, 8].

Epigenetic mechanisms, including DNA methylation, non-coding RNAs, and histone modifications, contribute to nongenetic adaptation, particularly in genetically uniform populations [9]. These processes are influenced by the microbiota, as shown in germfree models with altered methylation patterns [10] disrupted epigenetic regulation [11], and accelerated DNA methylation drift with age [12]. Microbial metabolites modulate epigenetic pathways [13], whereas host epigenetic processes can reciprocally influence microbial composition [14]. The microbiome’s ability to metabolise environmental compounds adds further complexity [15].

Carefully designed transplant experiments help untangle these interactions by introducing defined microbiota into different host genotypes under controlled conditions. Environmental context, such as diet, strongly influences transplant outcomes [16], though host genetic background can also constrain or redirect microbial assembly and function [17]. Integrative approaches linking host genomes to microbiome responses across environments are needed to move from correlations to causal understanding [18].

In dynamic freshwater ecosystems, microbiome-mediated adaptation may be especially important. *Daphnia magna* is a key ecotoxicology model due to its pollutant sensitivity [19, 20]. Its interactions with toxin-producing *Microcystis* species provide a framework for examining stress-induced microbiome shifts, which can lead to dysbiosis and reduced host performance [21–24]. *Microcystis* blooms, intensified by eutrophication and climate change, release microcystins that pressure *Daphnia*, drive coevolution [25], and may alter microbiota to aid detoxification [26, 27]. Moreover, *Microcystis* spp. are considered poor-quality food, lacking sterols and long-chain polyunsaturated fatty acids, containing γ-linolenic acid, potentially causing mechanical obstruction, and producing protease inhibitors [28–32].

DNA methylation is central in *Daphnia*’s response to stressors, with toxins, heavy metals, and cyanobacteria altering DNA methylation in genes linked to stress response, protein synthesis, and metabolism [33, 34]. For example, *Microcystis* toxins cause differential methylation of exonic regions, particularly at serine- and threonine-amino acid codons, and in genes for protein synthesis and transport, suggesting environmental modulation of DNA methylation [34]. High salinity induces hypomethylation in six protein-coding genes involved in DNA repair, cytoskeleton organisation, and protein synthesis, persisting for at least three generations [35]. Maternal dietary restrictions also influence DNA methyltransferase expression across generations [36], with methylation changes observed in unexposed offspring [37, 38]. Together, these findings position *Daphnia* as a promising model for epigenetic research in ecotoxicology [33, 34].

The *Daphnia* microbiome is shaped by host genotype, diet, and environment, with stressors altering its composition and potentially affecting host fitness [22, 26, 39–41]. This aligns with the holobiont framework, where hosts and microbiomes form integrated units (“holobionts”) with combined genomes (“hologenomes”) shaping host biology [42]. Host microbiomes can mediate tolerance to parasites and toxins, with locally adapted microbiomes enhancing stress tolerance [39, 43], though host genotype often plays an equal or greater role, especially in parasite resistance. This highlights the importance of considering both genetic and microbial ecological processes when interpreting host fitness outcomes [44–46].

We conducted a fully factorial reciprocal transplant with two *D. magna* genotypes to explore how gut microbiome and DNA methylation influence host performance under cyanobacterial stress. Microbiome-depleted juveniles were recolonised with microbiota from two genotypes, fed either toxic or nontoxic diets, while some remained uninoculated. Glutaraldehyde treatment was ineffective in one genotypes; therefore, results are presented for the effective genotype, with both genotypes included as donors. We assessed performance, gut microbial composition (16S rRNA gene sequencing), gut microbial functional profiling (WGS), and DNA methylation levels (colorimetric assays). We tested three hypotheses: (i) diet-shaped microbiomes influence host performance under toxin exposure; (ii) effects depends on host genotype; and (iii) performance changes may be mediated by epigenetic mechanisms such as DNA methylation. This study clarifies how gut microbiota interact with host genetics and epigenetics under environmental stress.

## Materials and methods

### Study organisms

Two *D. magna* genotypes or clones (B7 and B9), originally resting eggs-derived from a pond in Oud-Heverlee, Belgium [47], were maintained under standard laboratory conditions. The experimental diets consisted of the nontoxic green alga *Chlorella vulgaris* and the toxic microcystin-producing cyanobacterium *Microcystis aeruginosa* (strain PCC 7806). Full culturing protocols, UV treatment procedures (Table S1), and toxin quantification details are provided in Supplementary Methods.

### Experimental overview

**Figure 1:**
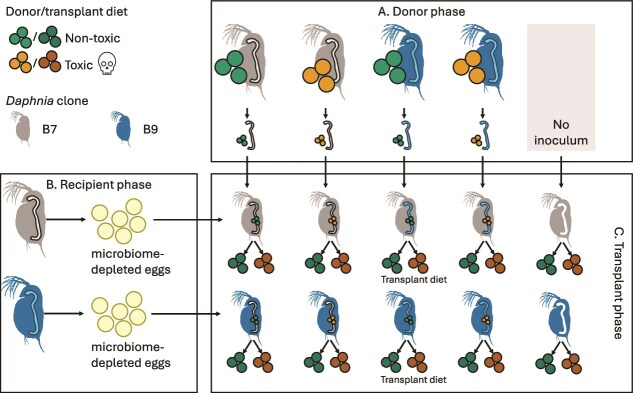
Overview of the experimental design. The diet consists of either *C. vulgaris* or *M. aeruginosa*, a distinction is made between donor and transplant diets in brighter and darker colours, respectively. (A) The donors (clones: B7 and B9) are presented in the top panel with their respective donor diets. (B) Gut microbiome depleted juveniles of the same clones are obtained as recipients, which are shown on the left. (C) The transplant phase, including their transplant diets and microbial inoculum (from donors), are presented in bottom right.

A full factorial transplant experiment was conducted investigating the effects of cyanobacterial stress on two clones of *D. magna*. These clones were used both to serve as gut microbiome depleted juveniles recipients and gut microbiome donors (Fig. 1). For each clone, three maternal lines were established by isolating individual females and rearing them separately. The experiment was conducted in three successive batches, each using one maternal line. Prior to the transplant, during the “donor phase”, 250 donor individuals per clone maternal line and diet combination were exposed for 21 days to either a toxic diet (a 50:50 mix of *M. aeruginosa* and *C. vulgaris*) or a nontoxic diet (*C. vulgaris* only). After this period, their guts were dissected to serve as microbiome donors, one gut served as donor for two juveniles in the transplant phase (Fig. S1). Simultaneously, in the “recipient phase”, 180 *Daphnia* per clone maternal line were maintained on the nontoxic diet. At the end of the 21-day period, ~720 eggs per clone maternal line were disinfected with glutaraldehyde to produce juveniles depleted of gut microbiota (Fig. S2). An additional group of juveniles did not receive any microbial inoculum.

Life-history traits (i.e. body size, total brood production, and survival) were monitored in both the donor and transplant phases. Body size of five adult *Daphnia* was measured at 0, 5, 10, and 15 days using stereomicroscopy and image analysis in the donor phase, and of 4 ± 1 adults in the “transplant phase” at day 9. Survival and brood production of five individuals were recorded over the 21 days of diet exposure in the donors. Survival percentage at 9 days in the transplant phase was assessed for 20 individuals per treatment in the first two maternal lines and 35 per treatment in the third. Microbial communities of five guts per treatment were characterised using 16S rRNA gene sequencing in the donor phase at 21 days of diet exposure, 15 whole animals in the recipient phase before and 15 juveniles after treatment, and the guts of 4 individuals per treatment in the transplant phase after 9 days of exposure. Global DNA methylation levels from 100 ng of DNA from 10 whole animals are also assessed in the transplant phase after 8 days. Shotgun metagenomic sequencing was performed on the same gut samples used for 16S rRNA gene sequencing from replicate 3 in the transplant phase. Full experimental procedures per phase and more information on the life-history assessments are provided in Supplementary Methods.

### Molecular analyses

DNA for 16S rRNA gene sequencing was extracted from *Daphnia* guts using the MasterPure Complete DNA and RNA Purification Kit, and libraries were prepared using a two-step PCR targeting the V4 region. Sequencing was performed on a MiSeq System (Illumina). DNA methylation levels on whole animals were quantified using the MethylFlash Methylated DNA Quantification Kit (Epigentek) following the manufacturer’s protocol. Whole-genome shotgun sequencing using a NovaSeq System (Illumina) (PE150, 3 Gb/sample) was conducted on the same DNA extracts from *Daphnia* guts as used for the 16S rRNA gene sequencing. Full protocols, including primer sequences, PCR conditions, and quality control steps, are provided in Supplementary Methods.

### Statistical analysis

#### Phenotypic endpoints

In the donor phase, we tested survival differences between donor clones (B7 and B9) and donor diets (nontoxic vs. toxic) using a mixed-effects Cox proportional hazards model, which accounts for random effects (maternal lines). A Gaussian General Linear Mixed Model (GLMM) assessed effects of donor clone, donor diet, time point, and their interactions on body size, with donor maternal line as a random effect. Simulated residuals confirmed model assumptions. Significant variables were identified by Type II Wald *χ*^2^ tests and post hoc least-squares means (BH correction [48]). The same model was applied to the final time point alone. Total brood size was analysed with a GLMM (nbinom1; V = μ(1 + φ) [49]) chosen by AIC. Fixed effects were donor diet, donor clone, and their interaction; maternal line was random. Simulated residuals confirmed model fit. Significant terms were identified by Type II Wald *χ*^2^ tests and post hoc least-squares means (BH correction [48]).

In the transplant phase, survival percentage was analysed with a Gaussian GLMM, restricted to recipient clone B9 due to sterilisation issues in B7 (see later). Fixed effects were donor clone, donor diet, transplant diet, and their interactions; maternal line was random. Simulated residuals indicated no overdispersion or assumption violations. Significant effects were identified by Type II Wald *χ*^2^ tests and post hoc least-squares means with BH correction [48]. A Gaussian GLMM assessed effects of donor clone, donor diet, transplant diet, and their interactions on body size, with maternal line as a random effect. Simulated residuals showed no assumption violations. Significant effects were identified by Type II Wald *χ*^2^ tests and post hoc least-squares means with BH correction [48].

#### Gut microbiota

After demultiplexing, 16S rRNA gene data were processed in QIIME2 (v2023.5), including denoising and ASV inference with DADA2 [50] and primer removal. ASVs were aligned using MAFFT [51], and a phylogenetic tree was built with FastTree [52, 53]. This tree was used to calculate Faith’s phylogenetic diversity and the weighted/unweighted UniFrac distances. Taxonomy was assigned using SILVA 138 (99% OTUs, full-length) [54–56], Greengenes2 2022.10 (full length) [55, 57], and Greengenes 13_8 (99% OTUs, 515F/806R region) [54, 55]. The ASV table, tree, and taxonomy were integrated into a phyloseq object, yielding 2368 ASVs.

Four filtering steps were applied: (i) removal of ASVs unassigned at the phylum level (60 ASVs, 2.5%); (ii) removal of ASVs where two classifiers failed to assign taxonomy more confidently than a third (76 ASVs, 3.2%); (iii) exclusion of six outlier ASVs by tree inspection; and (iv) removal of ASVs with conflicting phylum-level assignments across classifiers (24 ASVs, 1.0%), considering name harmonisation (e.g. TM6/*Dependentiae*). After filtering, 2277 ASVs remained across 79 samples (3.8% loss), with some ASVs flagged in multiple steps.

To minimise noise from low-abundance contaminants, ASVs present in only one sample were removed (Fig. S3), a crucial step given the absence of blank controls. After filtering, 392 ASVs remained (82.8% reduction in total number of ASVs), corresponding to an average per-sample loss of 25.7% of ASVs (95% CI: 24.0–27.4%) and 2.0% of reads (95% CI: 1.0–3.0%). Chloroplasts, mitochondrial, and cyanobacterial ASVs (11 ASVs) were also excluded. Rarefaction curves reached plateaus (Fig. S4). Averages from 100 rarefied datasets (depth = 6227 reads, lowest-read sample) yielded 381 ASVs across 79 samples.

This final phyloseq object was used to calculate diversity metrics for donor, recipient, and transplant phases.

Transplant fidelity was assessed using principal coordinate analysis (PCoA) on Bray–Curtis dissimilarity to visualise community structure. Within- and between-group dissimilarities were quantified for each maternal line using Bray–Curtis, and weighted/unweighted UniFrac dissimilarity metrics, where groups correspond to donor and transplant samples with the same microbial inoculum. Mann–Whitney tests were performed for each dataset and distance metric. Donor fidelity was further evaluated with PERMANOVA on the transplants to test the effects of microbial inoculum and maternal line, including their interaction, on all three distance metrics. Homogeneity of multivariate dispersion among groups was checked prior to PERMANOVA.

Alpha diversity (Faith’s phylogenetic diversity, Shannon Index, and species richness) was analysed using GLMMS. Faith’s and Shannon indices were normally distributed (confirmed by visual inspection and Shapiro–Wilk tests). Species richness was modelled using a Poisson distribution for the donor phase and a negative binomial (nbinom1, V = μ(1 + φ) [49]) for the recipient and transplant phases, based on AIC. Fixed effects included donor clone and diet (donor phase), recipient clone and microbiota-reducing treatment (recipient phase), and donor clone, donor diet, transplant diet, and their interactions (transplant phase); maternal line was random. Models excluding uninoculated samples included donor clone and diet as additional fixed effects. Significance was determined by Type II Wald *χ^2^* tests, with BH-corrected [48] post hoc pairwise comparisons.

Beta diversity was assessed using weighted/unweighted UniFrac and Bray–Curtis dissimilarities. PERMANOVA (1000 permutations, stratified by replicate) tested donor clone, donor diet, and transplant diet effects and their interactions. Homogeneity of dispersions was confirmed by permutation tests. Ordination used PCoA for each distance metric. Analyses were repeated for the donor phase (donor clone and diet) and the recipient phase (microbiota-reducing treatment and recipient clone).

Community-level dysbiosis was quantified as the median Bray–Curtis dissimilarity to a reference group of untreated recipient-phase samples [58].

#### Gut microbiota: functional profiling

Raw WGS reads from the third maternal line were quality-filtered using fastp v0.23.2 [59] to remove low-quality bases and adapters. The other maternal lines were excluded due to limited microbiota transfer, and resource constraints. Host-derived reads were identified by mapping against the *D. magna* reference genome (RefSeq ASM2063170v1.1, Table S2) [60] using Bowtie2 v2.4.4 [61], and separated into host and nonhost fractions with SAMtools v1.18 [62] and picard v2.18.23 [63].

Nonhost reads were assembled with MEGAHIT v1.2.9 [64] (default settings), and assembly quality evaluated using QUAST v5.2.0 [65]. Toxin-antitoxin loci were annotated via the Toxin-Antitoxin DataBase (TADB) 3.0 [66]; BLAST hits filtered in R v4.4.2 [67] with thresholds of ≥90% identity, *E* ≤1e^−5^, bit score ≥ 200, alignment ≥70% of the query, ≤ 5% mismatches, and ≤ 2 gap openings.

For broader functional profiling, nonhost reads were concatenated into single-sample FASTQ files and analysed using HUMAnN3 [68]. Gene family and pathway abundance tables were combined (humann_join_tables), normalised to counts per million (humann_renorm_table), regrouped to UniRef90 (humann_regroup_table), and annotated with MetaCyc reaction names (humann_rename_table).

Microbial functional variation was assessed using PCA on centred log-ratio (CLR) transformed data (mixOmics v6.30.0 [69]). Further analyses and plotting were conducted in R v4.4.2 [67] following previously published methods [70]. Associations with host performance (scaled to [0,1]) and DNA methylation were assessed using beta regression, with PCs (≥5% variance explained) as fixed effects. Model fit was evaluated via residual, leverage, and Cook’s distance diagnostics; moderately influential observations were retained unless they altered estimates. Pathways with loadings ≥0.25 for significant PCs were examined and visualised.

#### DNA methylation levels

Host DNA methylation percentages were tested for normality. As raw data deviated from normality (*W* = 0.92, *P* = .022), an arcsine square root transformation was applied, yielding normally distributed values (*W* = 0.95, *P* = .205), which were used in all analyses.

Effects of donor and transplant diet on DNA methylation in recipient clone B9 (B7 excluded due to sterilisation issues) were assessed with GLMMs with donor (clone and diet), transplant diet, and their interaction as fixed effects; plate number and maternal line as random effects. Plate number was included to account for two measurement runs. An additional model excluding uninoculated samples tested the three-way interaction between donor clone, donor diet, and transplant diet, with plate number and replicate as random effects. Simulated residual diagnostics indicated no assumption violations. Significance was determined via Type II Wald *χ^2^* tests, with post hoc least-squares means and BH correction [48].

#### Interactions between DNA methylation levels and phenotypic measurements

The relationship between host performance and DNA methylation was analysed using a multivariate mixed-effects model with Gaussian distribution, with scaled body size (day 9) and survival percentage normalised to [0, 1]. Fixed effects included DNA methylation percentage, donor (clone and diet), transplant diet, and their interactions; random effects used an unstructured covariance matrix to allow trait-specific intercepts and maternal line-level covariance.

A significant three-way interaction (DNA methylation × gut microbiome × transplant diet) led to separate models for each transplant diet, examining two-way interactions between donor (clone and diet) and DNA methylation. Simulated residual diagnostics indicated no major violations.

Predicted values and 95% CIs across the observed DNA methylation range were generated for each microbiome origin and used to plot model-predicted trends under nontoxic and toxic transplant diets.

All phenotypic, DNA methylation, and 16S rRNA gene analyses were performed in R v4.4.2 [67] using survival v3.7-0 [71], survminer v0.5.0 [72], coxme v2.2-22 [73], glmmTMB v1.1.10 [74], emmeans v1.10.7 [75], DHARMa v0.4.7 [76], ape v5.8-1 [77], phyloseq v1.50.0 [78], vegan v2.6-10 [79], and dysbiosisR v1.0.4 [80].

## Results

### Diet-shaped microbiomes influence host performance under toxic exposure in a host genotype-specific manner

Because the effects consistently interacted with host genotype, we address both hypotheses together. Results are presented in the following order: phenotypic measurements, microbiota composition from 16S rRNA gene sequencing (including dysbiosis), and microbiota functional profiling via WGS.

#### Phenotypic endpoints

The donor analysis showed that the toxic diet reduced body size (*χ^2^* = 6.1, *P* = .014) and total brood production (*χ^2^* = 19.6, *P* < .001), regardless of clone, whereas longevity was unaffected (Fig. S5, Table S3). On day 15, *Daphnia* from clone B7 were significantly larger than those from B9 on a nontoxic diet (t ratio = 2.3, *P* = .025) (Fig. S5B, Table S3B).

**Figure 2:**
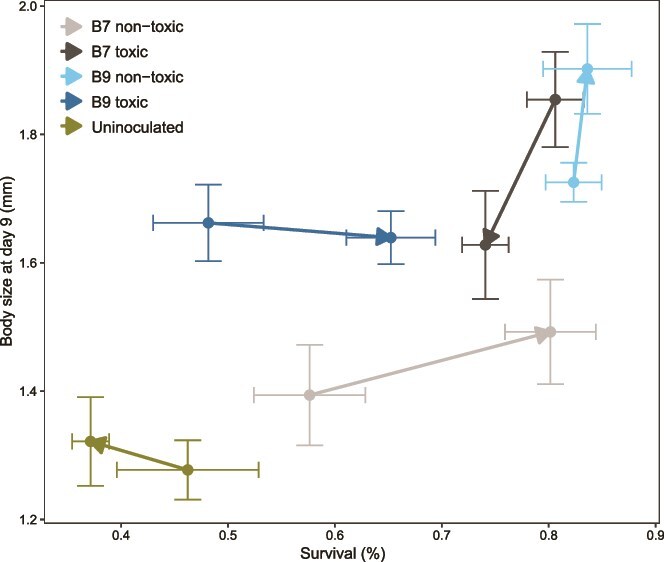
Transplant life history traits. The survival percentage is given on the x-axis, whereas the body size at day 9 (mm) is shown on the y-axis. The colours indicate the donor clones and donor diets. The point is the mean survival and body size, and the solid lines indicate the standard errors. The arrow starts from the nontoxic transplant diet and goes toward the toxic transplant diet.

After the transplants of the microbial inocula, we found that survival rates (*t* ratio = 3.5, *P* = .020 for B7C; *t* ratio = 4.3, *P* = .003 for B7M; *t* ratio = 5.0, *P* < .001 for B9C) and body sizes (*t* ratio = 6.9, *P* < .001 for B7M; *t* ratio = 8.2, *P* < .001 for B9C; *t* ratio = 5.5, *P* < .001 for B9M) were generally higher when a bacterial inoculum was present. Exceptions included survival in B9M (*t* ratio = 2.2, *P* = .213) and body size in B7C (*t* ratio = 2.2, *P* = .187), where no significant differences were observed compared to uninoculated *Daphnia* (Fig. 2 and S6, Table S4). *Daphnia* receiving the B7M inoculum were significantly smaller on the toxic diet than the nontoxic diet (*t* ratio = 2.7, *P* = .009), whereas those with the B9C inoculum were significantly larger on the toxic diet (*t* ratio = −2.2, *P* = .033) (Fig. S6B, Table S4).

Body size and survival percentage in the transplants were visualised together across donor clones and diets (Fig. 2). In general, survival increased with body size, but this varied by treatment. As previously described (Fig. S6 and Table S4), uninoculated groups showed both lower survival and smaller body size compared to all inoculated treatments. Additionally, individuals with a B9 donor clone exposed to a toxic donor diet had reduced survival compared to those from a nontoxic donor diet. In contrast, individuals with a B7 donor clone had smaller body sizes when fed a nontoxic donor diet compared to a toxic one. The previously reported significant differences in body size between transplant diets for B7M and B9C (Fig. S6B, Table S4) are again evident. No other pairwise comparisons between transplant diets were significant, and no significant differences in survival percentage were observed.

#### Gut microbiota

16S rRNA gene data indicated that the microbiota-reducing treatment was ineffective for recipient B7, as alpha diversity did not differ between treated and untreated samples (Faith’s PD: *t* ratio = −0.4, *P* = .700; species richness: *t* ratio = −0.8, *P* = .435; Shannon Index: *t* ratio = −0.9, *P* = .397; Fig. S7, Table S5). Although the microbiota-reducing treatment affected beta diversity overall (Fig. S8), interactions with recipient clone were significant for unweighted UniFrac (*F* value = 1.6, *P* = .036) and Bray–Curtis (*F* value = 3.8, *P* = .028), and marginal for weighted UniFrac (*F* value = 3.6, *P* = .059; Table S6). Therefore, only results from recipient B9 are presented, and B7 served only as a donor.

**Figure 3:**
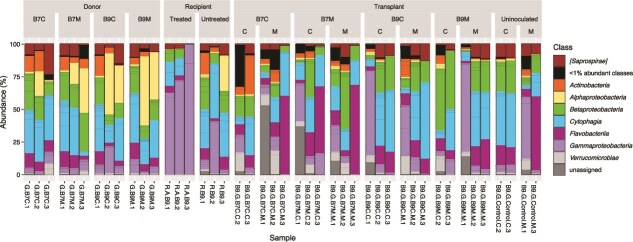
Bar plots of the microbial communities. Bar plots showing the relative abundance of different bacterial classes in the *Daphnia* gut microbiota. Results are shown for donors, recipients, and transplants. Not abundant classes are grouped within the <1% abundant classes. ASVs that were not assigned at class level are shown as unassigned.

The microbiota-reducing treatment altered microbial composition, with a relative increase in Gammaproteobacteria (Fig. 3). Successful microbial inocula transplants only occurred in the third maternal line (Fig. S9 and S10; Table S7), which was also used for WGS, whereas fidelity was low in lines 1 and 2. Differences were more pronounced with Bray–Curtis dissimilarity, which ignores phylogeny.

During the transplant phase, a significant donor × transplant diet interaction affected species richness (*χ^2^* = 9.8, *P* = .044), with donor B9M showing higher richness under the nontoxic diet (*z* value = 2.9, *P* = .004; Fig. S11, Table S8). Whereas no overall differences for beta diversity were found, excluding uninoculated samples revealed donor clone effects on beta diversity in weighted UniFrac (*F* value = 1.0, *P* = .048) and unweighted UniFrac (*F* value = 1.2, *P* = .027; Fig. S12, Table S9).

No significant effects on alpha or beta diversity were found among donor samples (Fig. S13, Tables S10–S11).

**Figure 4:**
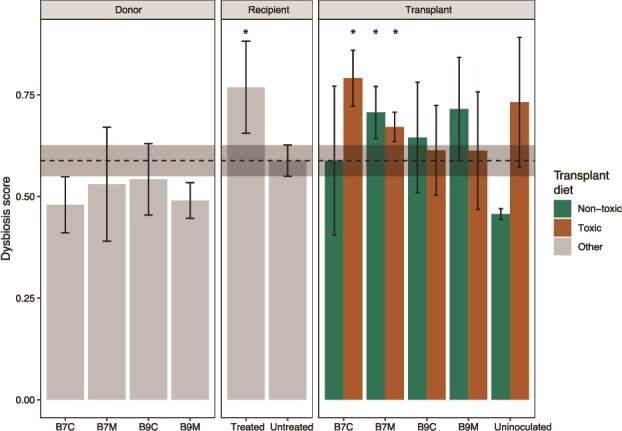
Dysbiosis scores**.** The dysbiosis score on the y-axis, based on Bray–like dissimilarities [58], measures how much a sample’s microbial composition deviates from the untreated recipients, which we considered as a healthy gut microbiome as they were not placed on a specific diet and untreated (indicated with the grey ribbon and dashed line). A lower score indicates a gut microbiota profile approximating healthy individuals, suggesting a more balanced microbial community. In contrast, a higher score reflects greater divergence from the healthy reference, implying a more dysbiosis or imbalanced microbial state (indicated with an asterisk on top). The different panels show the donor, recipient, and transplant phase, whereas the colours indicate the transplant diet. The x-axis shows the different treatments. Only results from B9 recipient clone are analysed as the microbiota-reducing treatment was ineffective for recipient clone B7.

Treated recipients exhibited higher dysbiosis scores. In the transplant phase, *Daphnia* inoculated with B7 microbiota showed elevated dysbiosis, except when both donor and transplant diets were nontoxic (Fig. 4).

#### Gut microbiota: functional profiling

**Figure 5:**
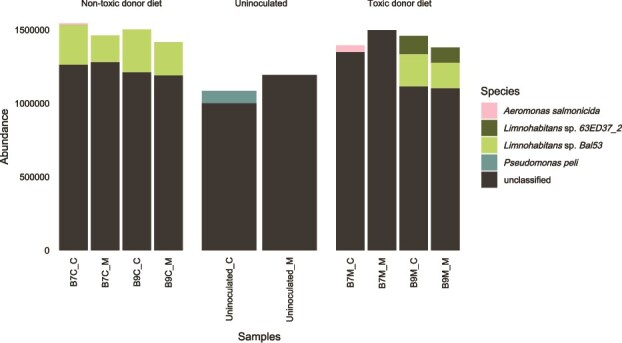
Functional profiling species. The abundance per functional pathway is given for which species are provided (*Aeromonas salmonicida*, *Limnohabitans* spp., and *Pseudomonas peli*) or unclassified. Unclassified species indicate that pathways are found but could not be assigned to a specific species. The samples are grouped per donor diet (nontoxic donor diet, toxic donor diet, and group without microbial inoculum) and the labels are showing the donor (donor clone and donor diet) followed by the transplant diet (C: nontoxic *Chlorella*, M: toxic *Microcystis*).

Taxonomic profiling using both TADB and HUMAnN3 consistently identified *Limnohabitans* spp. and *Pseudomonas* sp. as dominant species, with *Aeromonas* sp. additionally detected at lower abundance in HUMAnN3 (Fig. 5 and S14). Functional pathways attributed to *Limnohabitans* spp. were absent in *Daphnia* colonised with microbiota from the B7M donor and the uninoculated group (Fig. 5). Although *Limnohabitans* spp. genes were present in B7M samples according to TADB, they were at lower levels and entirely absent in the uninoculated group (Fig. S14).

**Figure 6:**
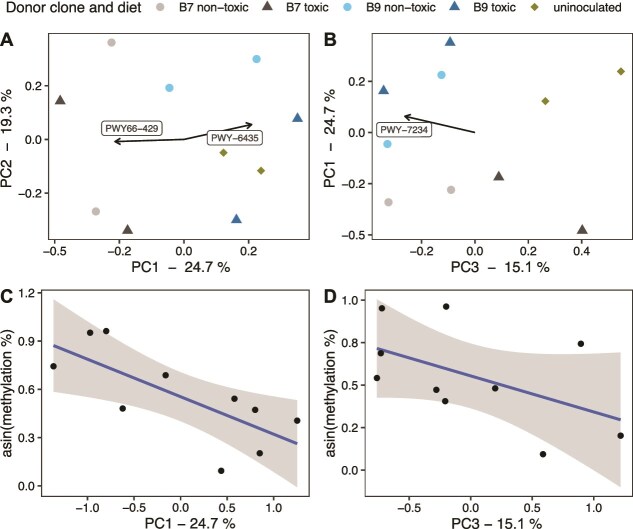
Functional profiling PCAs using coverage pathways (A and B). The PCAs are generated using code adapted from a previously published method [70]. For each sample, the PCA reflects variation among samples in both which pathways are present and how completely the pathways are covered (a threshold is used where only pathways that are covered for more than 50% are considered). Only pathways with PC loadings of ≥0.25 are visualised. *Daphnia* host mapped reads are removed in this analysis. DNA methylation on coverage pathways (C and D). The PCs where DNA methylation levels were significantly correlated with are presented. Combining both plots may indicate a potential positive correlation between DNA methylation levels and PWY66-429 (fatty acid biosynthesis initiation) and PWY-7234 (inosine-5′-phosphate biosynthesis III), and a negative correlation with PWY-6435 (4-hydroxybenzoate biosynthesis III) (more information on pathways in Table S12).

The transplant diet had minimal impact on pathway abundance but more noticeably influenced pathway coverage. Several principal components derived from functional abundance data were correlated with host performance and DNA methylation; however, no individual pathways exhibited loadings ≥0.25 (Fig. S15 and S16). In contrast, pathway coverage analysis revealed multiple associations with host performance. In particular, PWY66-429 (mitochondrial fatty-acid biosynthesis initiation) was positively associated with performance, whereas several pathways including PWY-6435 (4-hydroxybenzoate biosynthesis III), PWY-7220 (adenosine nucleotides de novo biosynthesis II), PWY-7222 (guanosine nucleotides de novo biosynthesis II), PWY-7221(guanosine ribonucleotides de novo biosynthesis), PWY-5695 (inosine 5′-phosphate degradation), PWY-5103 (L-isoleucine biosynthesis III), and ILEUSYN-PWY (L-isoleucine biosynthesis I) showed negative associations (Fig. S17). For DNA methylation, we observed that PWY66-429 (mitochondrial fatty-acid biosynthesis initiation) was positively associated, whereas PWY-6435 (4-hydroxybenzoate biosynthesis III) displayed a negative relationship (Fig. 6, pathways and functions summarised in Table S12).

### Performance changes are potentially mediated by epigenetic mechanisms such as DNA methylation

#### DNA methylation levels

**Figure 7:**
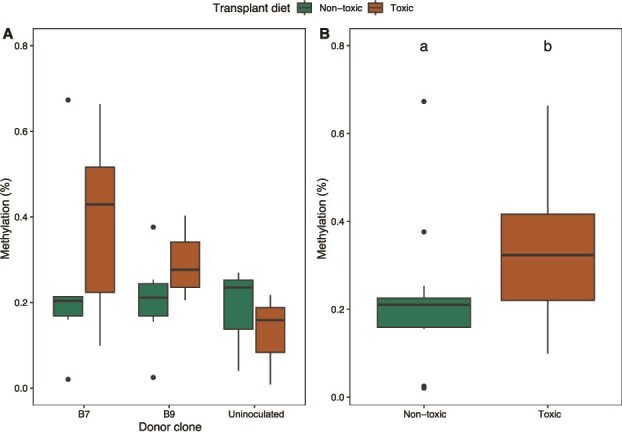
DNA methylation levels in the transplant phase. (A) Global DNA methylation levels are shown as the percentage of methylated cytosines (5-mC) relative to total DNA in each sample (i.e. 100 ng). Percentages were calculated from colorimetric absorbance values using the MethylFlash Methylated 5-mC DNA quantification kit (Epigentek) with positive and negative controls to establish a baseline. Higher percentages indicate a greater proportion of methylated DNA in the sample. The DNA methylation percentage ranging from 0.01% to 0.67% is shown per donor clone, and the colours indicate the transplant diet (the nontoxic *Chlorella* or the toxic *Microcystis*). We found a marginally significant higher DNA methylation level for the *Daphnia* with donor clone B7 compared to the treatment without donor inoculum when fed with a toxic diet. (B) The same data are presented as in A without the uninoculated treatment. The x-axis indicates the distinction between a nontoxic and a toxic transplant diet. A significant higher DNA methylation level is found under a toxic transplant diet.

No significant differences in DNA methylation were found when *Daphnia* received a nontoxic transplant diet (Fig. 7, Table S13). Under the toxic transplant diet, marginally significant increased methylation was observed for donor clone B7 compared to the uninoculated group (*t* ratio = 2.8, *P* = .051) (Fig. 7, Table S13). Overall, *Daphnia* receiving any donor inoculum showed significantly higher methylation levels on the toxic diet compared to the nontoxic diet, regardless of inoculum identity (*χ^2^* = 4.1, *P* = .042) (Fig. 7C, Table S13).

#### Interaction between DNA methylation levels and phenotypic measurements

**Figure 8:**
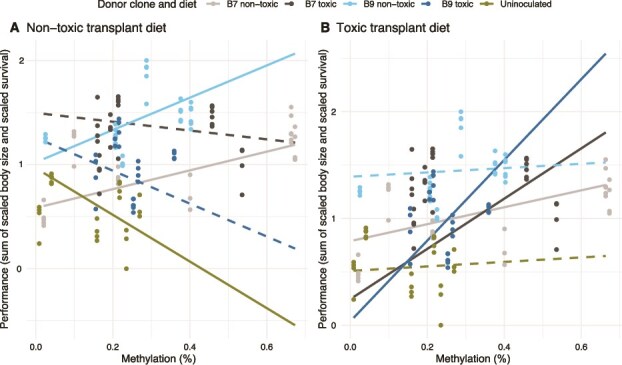
Interaction between life history traits and DNA methylation levels in the transplant phase. The sum of the scaled body size on day nine and the survival percentage is given as performance on the y-axis against the DNA methylation percentage on the x-axis. The points are the raw data, and the slopes are obtained from the multivariate random effects model. The solid lines indicate a significant slope, whereas the dashed lines are not significant. The results for the nontoxic transplant diet are shown in **A**, and the toxic transplant diet in **B**.

When placed on a nontoxic transplant diet, the only significantly positive DNA methylation-performance relationships were found in the nontoxic donor diets for both donor clones, B7 and B9. The slope for donor B7C was 0.9 (95% CI: 0.5 to 1.3), and for donor B9C it was 1.6 (95% CI: 0.8 to 2.3), both significantly different from the group without donor inoculum, which showed a significant negative trend of −2.2 (95% CI: −3.4 to −1.1) (Fig. 8A, Table S14).

When placed on a toxic transplant diet, significant positive slopes were mainly found in the toxic donor diets. Donor B7M had a slope of 2.4 (95% CI: 1.7 to 3.0), and donor B9M had an even steeper slope of 3.8 (95% CI: 2.2 to 5.3), both significantly different from all other groups (Fig. 8B, Table S14). The nontoxic donor diet from B7 (B7C) also remained significantly positive under the toxic transplant diet, with a slope of 0.8 (95% CI: 0.4 to 1.2), though it was significantly less steep than the slopes observed in the toxic donor diets.

## Discussion

Our study reveals that *D. magna* performance under toxic dietary stress is shaped by interactions among gut microbiota, host genotype, and epigenetic modifications. Donors fed a nontoxic diet exhibited better growth and reproduction than toxin-exposed donors, and absence of microbial inoculation reduced growth and survival, underscoring the importance microbes. The effect of microbiota pre-exposure to toxins depended on host genotype. Dysbiosis occurred when microbiota were transferred between nonmatching donor-recipient clones, and performance was linked to whether *Limnohabitans* spp. were present. Reduced performance on a toxic diet with microbiota from toxin-exposed, nonmatching clones suggests maladaptive microbial associations can arise under stress. Increased DNA methylation under toxic conditions was observed only when a microbial inoculum was present, emphasising the microbiome’s role in modulating host epigenetic responses.

The microbiome’s impact varied between host genotypes. Performance was particularly impaired when microbiota came from nonmatching clones, often showing dysbiosis and reduced presence of *Limnohabitans* spp. under a toxic donor diet. Contrary to our hypothesis, pre-exposure to toxins did not enhance growth in mismatched conditions; *Daphnia* were smaller under toxic diets. This could be due to the dysbiotic shift or a possible reduced adaptive potential instead of a pre-adaptation, resulting in a worse performance when placed again on a toxic diet. An exception occurred in *Daphnia* colonised with microbiota from the nonmatching donor-recipient clones that had never been exposed to the toxic diet, neither during the donor phase nor during the transplant. In these cases, the microbiota did not induce dysbiosis, indicating that both host genotype mismatch and prior stress contribute to maladaptive host–microbe interactions. In contrast, *Daphnia* receiving microbiota from matching clones fed a nontoxic diet grew larger on a toxic diet than on a nontoxic one. This pattern aligns with hormesis, where low toxin levels trigger adaptive responses that enhance growth, and is previously observed in *Daphnia* after a single exposure but not after repeated exposures [81]. Similarly, in our study, hormetic responses occurred only in *Daphnia* without prior exposure, those indirectly pre-exposed via microbiota showed no such effect. The negative impact of pre-exposed microbiota was absent when donor and recipient clones matched.

Previous studies show that matching donor-recipient clones enhances growth and reproduction, whereas mismatches often reduce fitness under stress [44, 82], supporting host-microbiota coadaptation where long-term associations foster mutual tolerance [45, 83]. Genotype-specific performance is also evident under environmental challenges like hypoxia [40], and priority effects during microbiome assembly vary by genotype and influence survival and fecundity [82]. Although not all studies find direct genotype effects on pathogen resistance [84], the overall evidence supports a synergistic role of host genotype and microbiota in shaping *D. magna* performance.

We found that DNA methylation may mediate responses to microbiota and environmental stress, reflecting both host and microbial DNA due whole-organism extraction. Observed methylation levels fall within reported ranges for *Daphnia* spp. [85] and *Arthropoda* [86]. Under nontoxic conditions, methylation levels did not differ between *Daphnia* with or without microbiota, suggesting microbiota alone do not alter DNA methylation without stress. Under toxic diets, methylation levels increased only in individuals receiving inocula, showing that microbial presence modulates epigenetic responses. Prior studies report genotype-dependent methylation changes in *Daphnia* exposed to *Microcystis* [87], suggesting context-dependent epigenetic regulation, potentially amplifying host stress responses. Alternatively, limited bacterial diversity in uninoculated treatments may reduce methylation potential. A marginal increase in methylation was also observed in *Daphnia* with nonmatching donor-recipient clones compared to the uninoculated individuals under toxic conditions. Moreover, methylation changes were possibly associated with mitochondrial fatty acid biosynthesis (PWY66-429, Table S12), consistent with evidence that microbiota-derived metabolites, such as short-chain fatty acids (SCFAs), influence host DNA methylation [88, 89] and can be affected by dietary shifts [90].

Our findings reveal context-dependent interactions between DNA methylation levels and *D. magna* performance, supporting a role for epigenetic regulation in environmentally responsive phenotypic plasticity. Under nontoxic transplant conditions, positive correlations between DNA methylation and performance were observed only in individuals from nontoxic donor diets, whereas uninoculated individuals showed a negative relationship, suggesting that the absence of microbiota disrupts adaptive DNA methylation–performance coupling. These results align with previous studies demonstrating that DNA methylation can be adaptively tuned to specific environmental histories and diets [91, 92]. Under toxic transplant diets, strongest positive correlations occurred in individuals from toxic donor diets. This suggests that epigenetic modifications induced by toxic environments may prime the host for improved physiological tolerance, likely through immune or detoxification gene regulation. Overall, these results support the view that DNA methylation may mediate plastic responses in an environment-dependent manner, consistent with ecological epigenetics frameworks [93]. Future work on specific methylated loci could clarify which methylation changes are adaptive.

The genus *Limnohabitans* appears to support *D. magna*’s tolerance to toxic cyanobacterial diets. *Daphnia* lacking this genus, either uninoculated or colonised with microbiota from nonmatching donor-recipient clones previously exposed to toxins, showed reduced performance on toxic diets. High *Limnohabitans* spp. abundances in the *Daphnia* gut are associated with enhanced survival, growth, and reproduction under microcystin-producing *Microcystis* exposure [22, 94], likely due to beneficial metabolic functions such as nutrient assimilation or detoxification. It is thought that *Limnohabitans* is a keystone genus in the *Daphnia* microbiome, especially in ecotoxicological contexts [95, 96].

Although transplant fidelity was relatively low, *Daphnia* were sampled after eight days in the host, allowing microbial communities to shift and adapt to host and environment. Fidelity appeared higher with a nonphylogenetic beta diversity metric, suggesting that although community composition changed, the taxa remained closely related, which is unsurprising given that only two *Daphnia* populations were involved. This pattern is further supported by the transplant microbiota, where the donor clone continued to shape microbial composition.

## Conclusion

Our findings indicate that host performance under environmental stress depends on microbiota identity and compatibility, host genotype, and their dynamic interplay, potentially mediated via epigenetic plasticity. They highlight the importance of integrating microbial, genetic, and epigenetic perspectives in studies of ecologically relevant organisms. This holobiont system offers a framework for future research, which is increasingly relevant as anthropogenic stressors impact aquatic ecosystems.

## Supplementary Material

Daphnia_toxic_methylation_Supplementary_ISME_v2_wraf247

## Data Availability

The datasets generated during and/or analysed during the current study are available in the Zenodo repository, [10.5281/zenodo.15831762] and NCBI Sequence Read Archive under BioProject accession [PRJNA1284238].

## Author information

Conceptualisation: NG, JA, ED. Methodology: NG, JA, ED. Investigation: NG, MC, IV. Formal Analysis: KB. Visualisation: KB. Writing - Original Draft: KB. Writing & Editing: KB, NG, MC, IV, AG, JA, ED. Generative AI was used to refine the manuscript’s written text.
