# Supplementary material for: Host-microbiota matching and epigenetic modulation drive Daphnia magna responses to cyanobacterial stress
Source: ISME J. 2025 Nov 3;19(1):wraf247. doi: 10.1093/ismejo/wraf247 (PMC12642672; doi:10.1093/ismejo/wraf247)
Supplement: Daphnia_toxic_methylation_Supplementary_ISME_v2_wraf247 [file daphnia_toxic_methylation_supplementary_isme_v2_wraf247.pdf]

Supplementary information: Host-microbiota matching and epigenetic modulation drive *Daphnia magna* responses to cyanobacterial stress.

SUPPLEMENTARY METHODS

Study Organisms

The B7 and B9 genotypes of *Daphnia magna* Straus (1820) used were originally derived from resting eggs recovered from the bottom sediment of an 8.7 ha fish culture pond established in 1970 in Oud-Heverlee, Belgium (Cousyn et al., 2001 in PNAS). The genotypes are maintained in the Laboratory of Aquatic Ecology at KU Leuven Kortrijk (KULAK) at a 16:8 light-dark cycle, 20°C, and are fed three times a week with frozen *Chlorella vulgaris* (200 × 10^3 cells/mL). No sterilization treatment was performed for feeding the stock cultures. To minimize maternal effects within each genotype, three maternal lines were established per genotype by propagating every second (or third) brood over at least two generations. These genotypes are hereafter referred to as clones.

The experimental diet consisted of the green microalga, *Chlorella vulgaris,* and the toxic cyanobacteria, *Microcystis aeruginosa*. *M. aeruginosa* strain PCC 7806, with verified microcystin production, was isolated from the Braakman reservoir in the Netherlands and is part of the Culture Collections at the Institut Pasteur (Paris, France). *C. vulgaris* and *M. aeruginosa* were cultivated in Wright's Cryptophyte (WC) medium and modified WC medium (without Tris), respectively. The microalgae and cyanobacteria were maintained under sterile conditions in a climate-controlled chamber at 22 ± 1 °C, following a 16:8 h light-dark cycle, in 2 L glass bottles with continuous stirring and aeration. To prevent bacterial contamination, 0.22 µm filters were installed at both the inlet and outlet of the aeration system. The algae and cyanobacteria were concentrated by centrifuging in a 50 mL falcon for 10 min at 3500 rpm. Besides, for the transplant diet, UV exposure was implemented by placing the falcon tubes containing the algae and cyanobacteria under UV light in a laminar flow hood for 30 minutes [1]. Samples were taken for MC-LR before and after UV treatment to analyse the effect of the treatment and to measure the toxicity of *Microcystis aeruginosa* in collaboration with De Watergroep. Given that the experiment was conducted in three separate batches, one per maternal line, 50 mL of cyanobacteria were prepared per batch in Falcon tubes (Table S1). The average toxin level was 449.33 ± 34.95 µg/L before UV treatment and 415.33 ± 54.93 µg/L after UV exposure. Our measured toxin levels were relatively high (between 0.3 and 15 µg/L in Sabart *et al.*, 2010; between 6 and 12 µg/L in Ait Hammou *et al.*, 2014) but not unreasonably high during blooms (between 1300 and 1800 µg/L in Jones & Orr, 1994).

Phenotypic measurements: body size, total brood size, and survival

Body size measurements were obtained using a stereomicroscope (Olympus SZX16) equipped with a BMS camera (Bennink Meijer Specialized). Images were processed using ImageJ (version 1.53J), and body size was measured as the length from the top of the head to the base of the apical spine. In the donor phase, the body size of five randomly selected individuals from each treatment in the 2-L jars was measured on days 0, 5, 10, and 15. During the transplant phase, on day 9, body size was measured for four individuals per treatment, except in cases where fewer than four individuals survived.

Additional life-history traits, including total brood size, and survival, were monitored in the separate 250-mL controlled jars during the donor phase. The survival of the initial five females and total brood production were recorded every other day over 21 days. Offspring were removed from the jars to avoid confusion with the original females. In the transplant phase, juvenile *Daphnia* were monitored for overall survival after eight days.

Donor phase

To assess survival and fecundity during the donor phase, a total of 180 *Daphnia* were used consisting of 15 *Daphnia* individuals (5 individuals x 3 250 mL jars with filtered tap water) per donor maternal line and diet combination (12 in total: 2 donor *Daphnia* clones x 3 maternal lines per clone x 2 diets).

To have sufficient donor gut inoculum, an additional 3000 *Daphnia* were used, consisting of 250 *Daphnia* individuals (50 individuals x 5 2L jars with filtered tap water) per donor maternal line and diet combination (12 in total: 2 donor *Daphnia* clones x 3 maternal lines per clone x 2 diets). The body size on days 0, 5, 10, and 15 was measured from randomly selected individuals from the 2L jars. *Daphnia* were fed three times per week with their designated diet. The non-toxic diet consisted of 2*105 cells/mL of *Chlorella vulgaris*, whereas the toxic diet was a mixture of 1*105 cells/mL of *Microcystis aeruginosa* and 1*105 cells/mL of *C. vulgaris*. The donor phase lasted for 21 days, after which the guts of the donor *Daphnia* were dissected. These gut samples were crushed with a sterilized pestle and homogenized in 100 µL of MQ water within 1.5-mL Eppendorf tubes, which were kept on ice during preparation. The inocula were used on the same day for the recipient *Daphnia*, except for half of the treatments in the first batch. Due to an insufficient number of gut microbiome depleted recipients, half of the inoculum from the first batch was stored at 4°C for later transplant three days later. One gut served as the donor gut microbiota inoculum for two *D. magna* juveniles during the transplant phase.

Recipient phase

To obtain the necessary gut microbiome depleted individuals for the recipient phase, a total of 18 500 mL jars with filtered tap water, each containing 10 *Daphnia magna* were set up per maternal line. These *Daphnia magna* individuals were fed daily with the non-toxic diet and to ensure optimal reproduction conditions, the experimental jars were refreshed when debris accumulated, or when populations became overly dense. The recipient phase lasted for 21 days, after which *Daphnia* females carrying eggs at the 12 to 24-hour developmental stage [38] were dissected under a stereomicroscope using dissection needles. Females carrying older eggs, identified by the absence of an external membrane, were excluded to enhance hatching rates after glutaraldehyde (GA) disinfection [38].

Approximately thirty eggs per well were placed in six-well plates containing 5 mL of filtered tap water, resulting in a total of about 720 eggs per maternal line per clone. This number was necessary to account for the 50% hatching rate. Under a laminar flow hood, the dissected eggs were transferred using sterile glass micropipettes to a six-well plate with 5 mL of 0.1% GA solution. After 10 minutes of gentle agitation, the eggs were moved to a new six-well plate containing 5 mL of sterile filtered tap water to remove GA residues. This washing step was repeated twice, after which the eggs were transferred to a final six-well plate with 5 mL of sterile filtered tap water. The plates were sealed with parafilm and incubated at 20°C for 72 hours under a 16:8 light-dark cycle to allow hatching (Fig. S2). The gut microbiome depleted juveniles obtained were subsequently used in the transplant phase of the experiment. To verify the sterility of these juveniles, fifteen individuals per clone were assessed. Additionally, before starting the microbiota-reducing treatment, fifteen whole individuals were collected without sterilization as positive controls.

Transplant phase

In the transplant phase, a total of 750 gut microbiome depleted *Daphnia magna* juveniles per clone from the recipient phase were transferred into petri dishes containing 45 mL of sterile filtered tap water under a laminar flow hood. The microbial inocula were then introduced by

adding one gut per two sterilized juveniles. The group without supplementary gut microbiota did not receive any gut microbiome inoculum. The petri dishes were sealed with parafilm to maintain sterile conditions and incubated at 20°C for 48 hours, allowing the *Daphnia* to take up the gut microbiota. After the incubation period, the contents of the petri dishes were transferred into 1-L jars containing 750 mL of sterile filtered tap water. These jars were equipped with specialized caps featuring ePTFE membranes with 0.2 µm pore size filters (Carl Roth), enabling air exchange and preventing contamination. The *Daphnia* were fed every other day under the laminar flow hood with their respective sterile diets: either the toxic or the non-toxic diet. The jars were stored in the laboratory at 20°C under a 16:8 light-dark cycle.

<u>16S rRNA gene sequencing – sampling, DNA extraction, library preparation, and sequencing</u>

In the donor phase, fifteen guts were dissected (Fig. S1) per treatment using two dissection needles under a stereomicroscope. This was done by opening the mandibles and pulling out the gut as intact as possible to preserve all gut microbiota. Samples were afterwards snap-frozen in liquid nitrogen and stored at -80°C until further processing. The same procedure was followed for the transplant phase, where four individuals per treatment were dissected.

To obtain DNA, we used the MasterPure Complete DNA and RNA Purification kit from Biosearch Technologies following the protocol of the manufacturer. The DNA was resuspended in 30 µL of DNase and RNase-free MQ. The quantity and quality of the DNA samples was assessed using an Implen Nanophotometer (model number: P-Class) spectrophotometer by evaluating the A260/280 and A260/230 ratios and the peak at 260 nm in the absorbance spectrum.

For library preparation, we performed an external PCR using universal eubacterial primers 27F (5’ – AGA GTT TGA TCM TGG CTC AG – 3’) and 1492R (5’ – GGT TAC CTT CTT ACC ACT T – 3’) (Weisburg et al., 1991). The PCR mixture consisted of 2.5 µL buffer, 0.75 µL dNTPs, 1.5 µL of each primer (10 µM), 0.2 µL Pfx DNA polymerase, and 10 ng of DNA template, adjusted to a final volume of 25 µL with sterile MQ water. The PCR cycling conditions included an initial denaturation of 3 minutes at 94°C. This was followed by 30 cycles of 30 seconds at 94°C, 45 seconds at 50°C, and 90 seconds at 68°C. The final extension was 10 minutes at 68°C with a cooling down to 12°C afterwards. We cleaned the DNA for next generation sequencing library construction using CleanNGS (CleanNA) following the manufacturer's protocol and assessed quality and quantity using an Implen Nanophotometer (model number: P-Class). The cleaned PCR product was then used in triplicate in an internal PCR targeting the V4 region with primers 515F_1 (5’ - AATGATACGGCGACCACCGAGATCTACACatcgtacgTATGGTAATTGTGTGCCAGCMGCCGCGGTAA) and 806R_1 (5’ - CAAGCAGAAGACGGCATACGAGATactatgtcAGTCAGTCAGCCGGACTACNVGGGTWTCTAAT) [39, 40]. The reagent composition for the internal PCR was 2.5 µL buffer, 0.75 µL dNTP mixture, 0.5 µL $Mg[SO]_4$, 0.75 µL of each primer, 0.2 µL Pfx DNA polymerase, 5 µL of template, and 14.55 µL of sterile MQ water. The PCR cycling conditions included 3 minutes at 94°C for initial denaturation, followed by 30 cycles of 30 seconds at 94°C, 30 seconds at 55°C, and 1 min at 68°C. The final extension was 10 minutes at 68°C. After pooling the three technical replicates, we performed a gel electrophoresis on a 1.5% agarose gel pre-stained with GelRed with 5 µL PCR product and 1 µL of loading dye alongside 3 µL of SmartLadder SF 100-1000 bp (Eurogentec). The PCR products were purified as described before (CleanNGS) and normalized using the SequalPrep Normalization Plate from Invitrogen before sending them to Genomics Core for 16S rRNA gene sequencing on a MiSeq V2 System (PE500) (Illumina).

DNA methylation levels – sampling, DNA extraction

In the transplant phase, whole animals were stored per treatment and clone at -80°C after snap-freezing in liquid nitrogen for quantification of the DNA methylation levels. The DNA was extracted using MasterPure Complete DNA and RNA Purification kit from Biosearch Technologies following the manufacturer's protocol. This resulted in 59 samples: two recipient clones, five different microbial inocula, two different transplant diets, and three replicates (one sample could not be processed due to a lack of surviving animals). From each sample 100 ng of DNA was loaded in triplicate to 96-well plates. Besides, two positive and two negative controls were added to ensure reliability of the assay results across larger batches of samples. These controls were also used to establish a baseline for DNA methylation detection.

To capture DNA for subsequent measurement of DNA methylation levels, 80 µL of Binding Solution (ME2) was added to each well to ensure high DNA affinity. This step was crucial for the assay's specificity, as it ensured that only DNA bound to the wells would proceed to the subsequent DNA methylation detection steps. Methylated DNA was captured using a capture antibody, followed by detection with a detection antibody. The quantification of methylated DNA was achieved colorimetrically by measuring absorbance at 450 nm using a microplate spectrophotometer. The absorbance was directly proportional to the amount of methylated DNA, allowing for the quantification of global DNA methylation levels in the samples.
All steps from the MethylFlashTM Methylated 5 mC DNA Quantification Kit (Colorimetric) (Epigentek) were conducted at the Bioengineering Faculty of Ghent University following the manufacturer's protocols, including recommended incubation times and temperatures.

Shotgun sequencing

The same DNA extractions used for 16S rRNA gene sequencing were sent to the KU Leuven Genomics Core for shotgun metagenomic sequencing. DNA quality and quantity were assessed using Nanophotometer. Libraries were prepared and sequenced using a NovaSeq System (Illumina) with 150 bp paired-end reads (PE150) by the KU Leuven Genomics Core, targeting a sequencing depth of 3 Gb per sample.

REFERENCES

1. Mehta R, Hawxby K. Use of Ultraviolet Radiation To Achieve Bacteria-Free Algal Culture. Proc Okla Acad Sci. 1977;57: 54–60. Available: https://ojs.library.okstate.edu/osu/index.php/OAS/article/view/5055/4725
2. Sabart M, Pobel D, Briand E, Combourieu B, Salençon MJ, Humbert JF, et al. Spatiotemporal Variations in Microcystin Concentrations and in the Proportions of Microcystin-Producing Cells in Several Microcystis aeruginosa Populations. Appl Environ Microbiol. 2010;76: 4750–4759. doi:10.1128/AEM.02531-09
3. Ait Hammou H, Latour D, Sabart M, Samoudi S, Mouhri K, Robin J, et al. Temporal evolution and vertical stratification of Microcystis toxic potential during a first bloom event. Aquat Ecol. 2014;48: 219–228. doi:10.1007/s10452-014-9477-0
4. Jones GJ, Orr PT. Release and degradation of microcystin following algicide treatment of a Microcystis aeruginosa bloom in a recreational lake, as determined by HPLC and protein phosphatase inhibition assay. Water Res. 1994;28: 871–876. doi:10.1016/0043-1354(94)90093-0

FIGURES

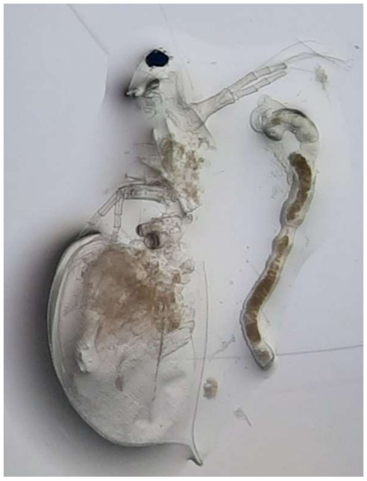

Figure S1: Gut dissection of *Daphnia magna*. On the right, the intact gut is shown, and on the left the leftover body tissues (picture taken by NG).

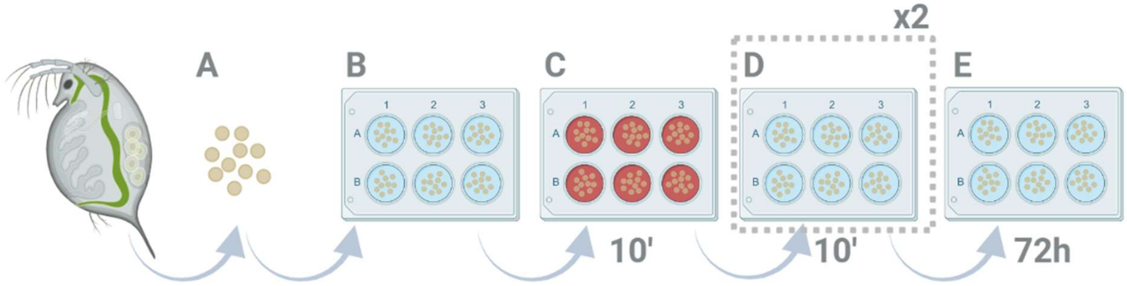

Figure S2: Microbiota-reducing *Daphnia magna* protocol (following Callens et al., 2016). A) Dissected *Daphnia magna* eggs in the 12–24-hour developmental stage. B) Dissected eggs are placed in a six-well plate with sterile filtered tap water. C) 10 minutes of agitation in 0.1% Glutaraldehyde (GA). D) Two washing steps with sterile filtered tap water to remove GA residues (10 minutes). E) Disinfected eggs are transferred to six-well plates with sterile filtered tap water and placed in an incubator for 72h to allow hatching of the animals. Figure is created using BioRender.

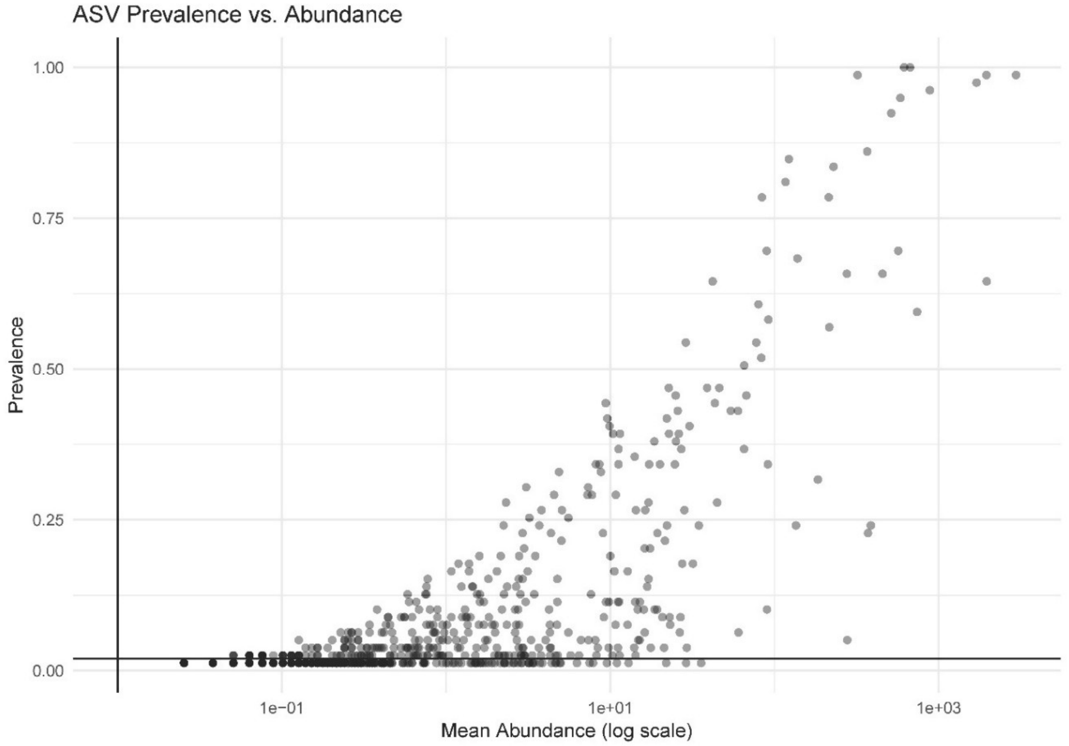

Figure S3: ASV prevalence versus ASV abundance. Each point is an ASV that was found in at least one of the samples. All ASVs below the horizontal line (prevalence of 0.02) were occurring in only a single sample and were removed from further analyses.

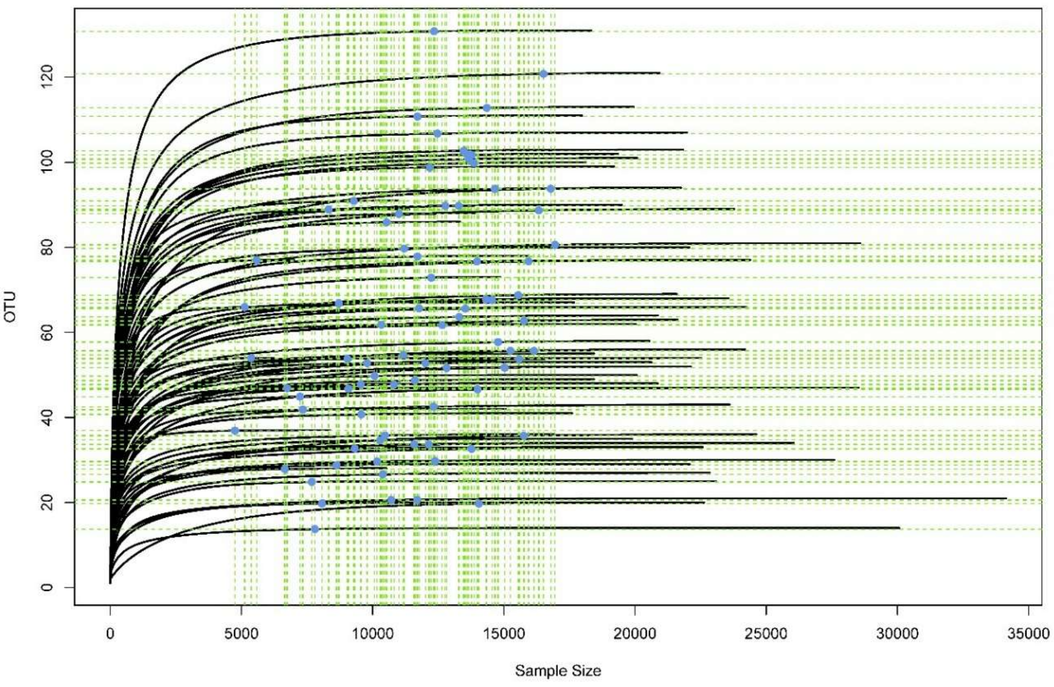

Figure S4: Rarefaction curves of the different samples. Rarefaction curves (threshold = 0.001) were made to ensure that samples reached plateau phase as shown here with the green dashed line.

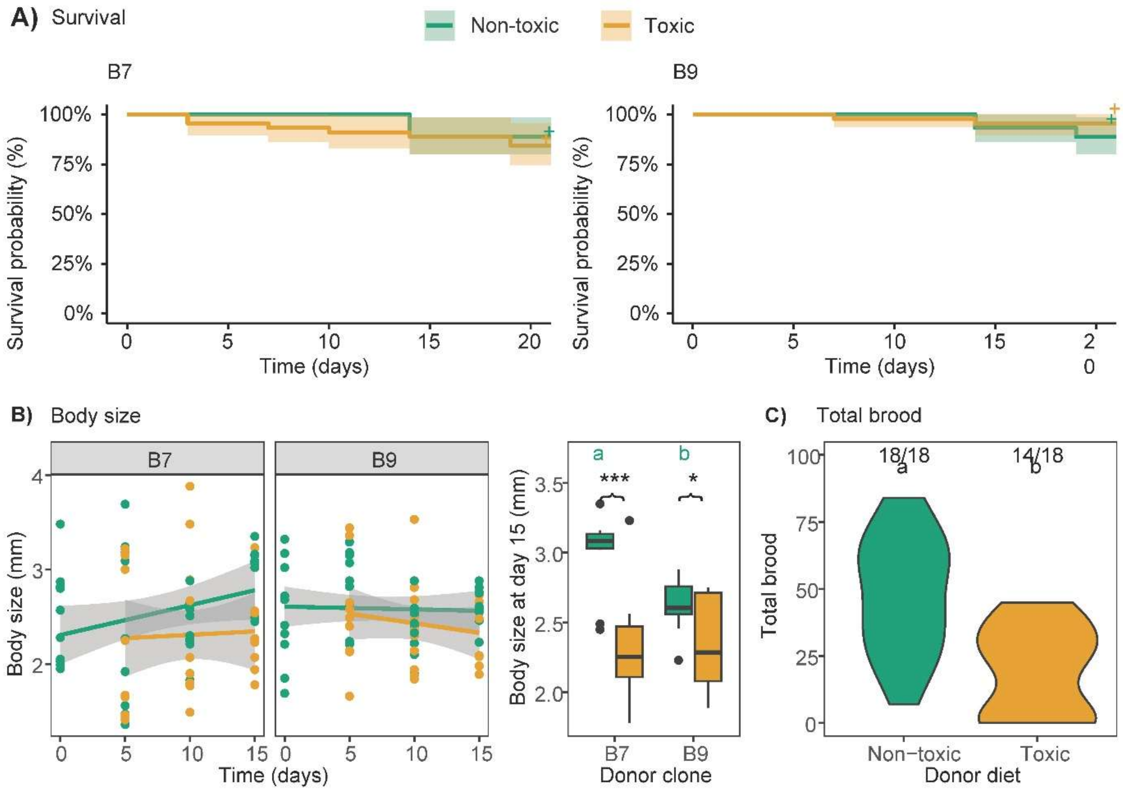

Figure S5: Donor phenotypes. A) Survival probabilities on the y-axis in time for the two donor clones (B7 and B9) and their diets (the non-toxic *Chlorella* in green and the toxic *Microcystis* in orange). No significant differences are found. **B)** Body size (mm) on the y-axis and time on the x-axis for the two donor clones B7 and B9 and their diets (the non-toxic *Chlorella* in green and the toxic *Microcystis* in orange). The body size is overall lower under the toxic diet, and after 15 days also a significant difference on the non-toxic diet between both clones is found where the *Daphnia* from donor clone B7 are larger than those from donor clone B9. **C)** The total brood size (i.e., the sum of the juveniles from 5 individuals per jar) on the y-axis and the donor diet on the x-axis (the non-toxic *Chlorella* in green and the toxic *Microcystis* in orange). The total brood size is significantly higher under a non-toxic diet as also indicated with the significance letters on top. The numbers on top indicate in how many of the jars juveniles were found.

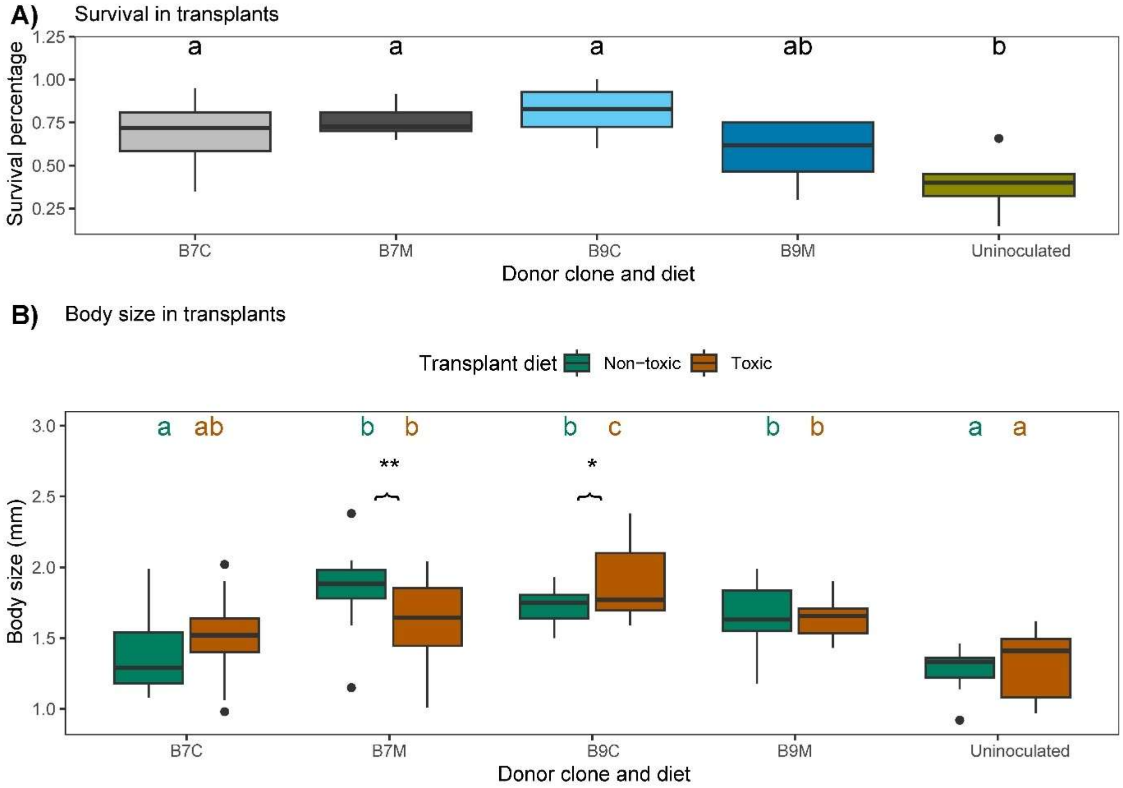

Figure S6: Transplant phenotypes. A) The boxplots show the survival percentage for each donor clone and diet on the x-axis. Only results for recipient clone B9 are presented due to problems with microbiota-reducing treatment in recipient clone B7. A lower survival was found in those *Daphnia* that did not receive an inoculum compared to those that received an inoculum, except for B9M. **B)** The boxplots show the body size (mm) per donor clone and diet, whereas the colours indicate the transplant diets (dark green for non-toxic and dark orange for toxic diets). The dark green significance letters on top indicate significant differences between donor clones and diets that obtained a non-toxic transplant diet, whereas the significance letters in orange show the difference for those that obtained a toxic transplant diet. The asterisks indicate significant differences within donor clone and diet between the transplant diets. All *Daphnia* were larger when they received a bacterial inoculum, except for B7C. *Daphnia* with a B7M inoculum were significantly smaller under a toxic diet than under a non-toxic diet, and the *Daphnia* with a B9C inoculum were significantly larger under a toxic diet compared to a non-toxic diet.

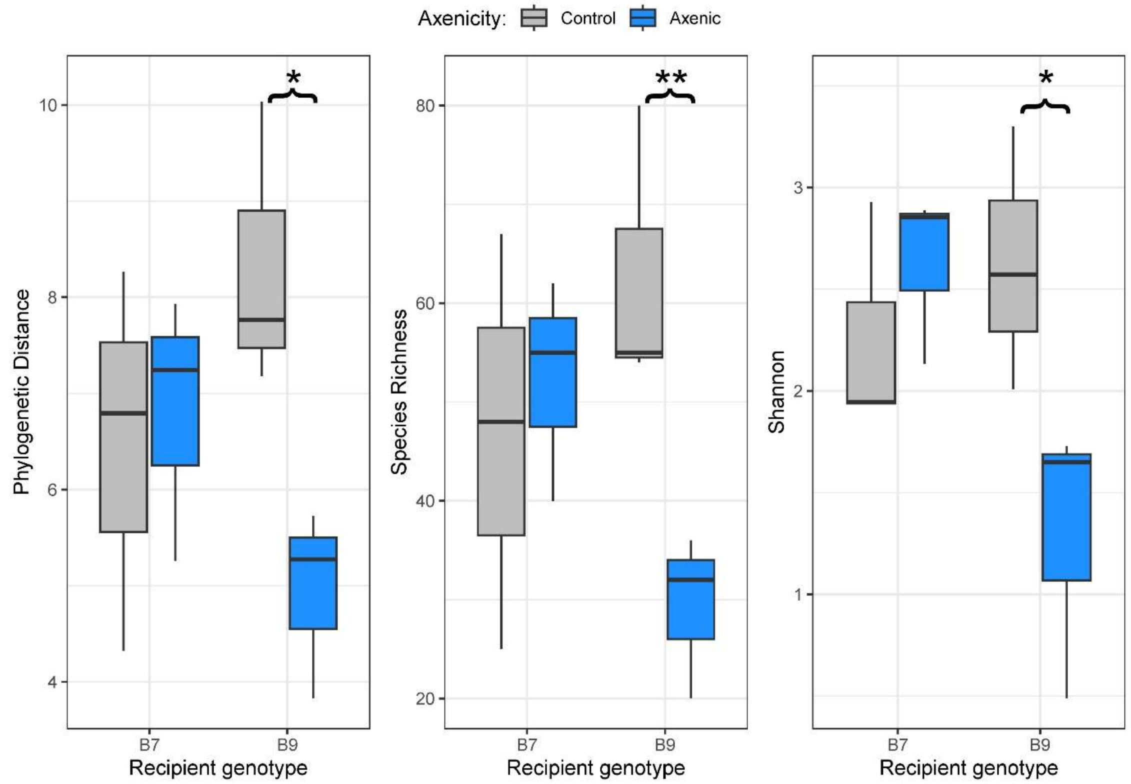

Figure S7: Alpha diversity in recipient phase. The y-axis shows the different alpha diversity metrics (i.e., Faith's phylogenetic distance, species richness and Shannon Index), whereas the different recipient genotypes (B7 and B9) are presented on the x-axis. In grey are the untreated *Daphnia* and in blue the *Daphnia* treated with glutaraldehyde to create microbiome depleted individuals. The treatment was only effective in recipient genotype B9.

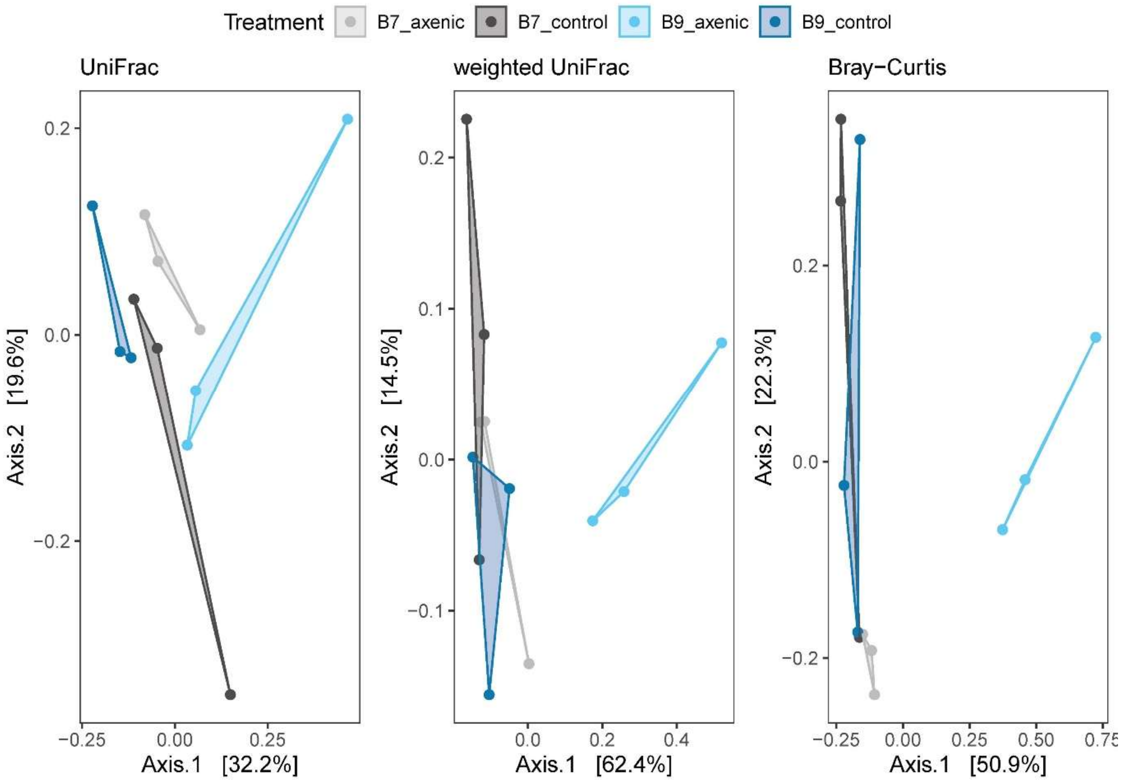

Figure S8: PCoA plots of the beta diversity in recipient phase. The different beta diversity metrics are shown as panels (i.e., unweighted UniFrac, weighted UniFrac, and Bray-Curtis distance). In grey is the recipient genotype B7 shown whereas blue is the recipient genotype B9. Brighter colours are the individuals treated with glutaraldehyde to obtain microbiome depleted individuals, and darker colours are the untreated individuals. Only genotype B9 shows a clear separation between treated and untreated individuals.

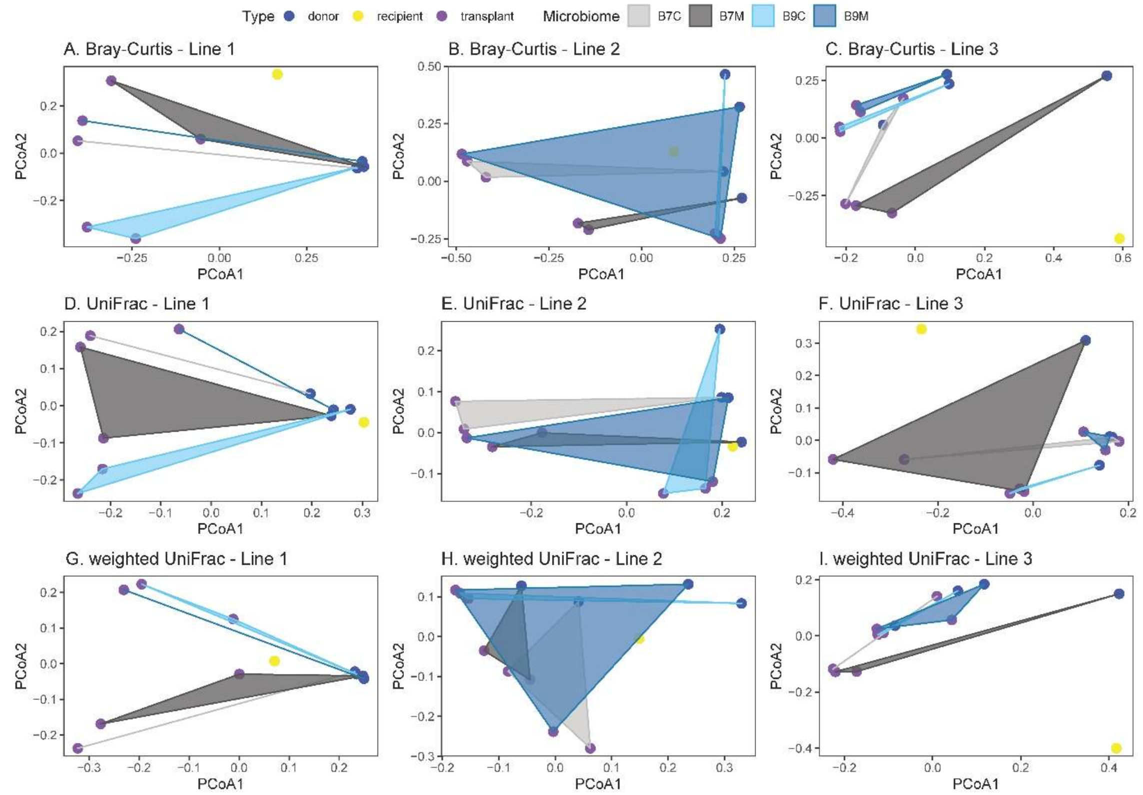

Figure S9: Fidelity of microbial transplants from donors in microbiota-depleted recipients. The different colours of the points represent the main type (i.e., donor in blue, recipient in yellow, and transplant in purple). The microbial inocula are shown as connecting polygons in their respective colours (B7C in light grey, B7M in dark grey, B9C in light blue, and B9M in dark blue), these are connecting the donors with their transplants. The different beta diversity metrics are presented per row (A-C: Bray-Curtis; D-F: unweighted UniFrac distance; G-I: weighted UniFrac distance), whereas the columns indicate the different maternal lines (A, D, and G: maternal line 1; B, E, and H: maternal line 2; C, F, and I: maternal line 3).

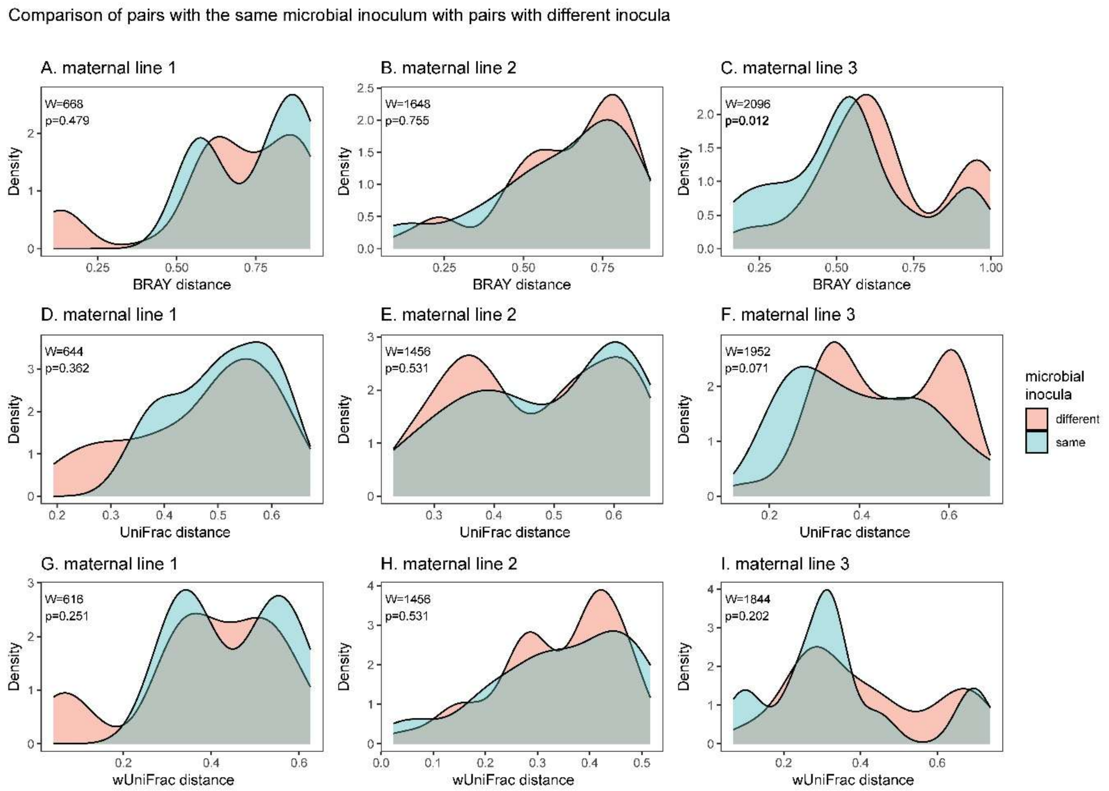

Figure S10: Density plots showing the comparison between two sample from donors or transplants with the same microbial inoculum (in blue) and different microbial inocula (in pink). The different beta diversity metrics are presented per row (A-C: Bray-Curtis; D-F: unweighted UniFrac distance; G-I: weighted UniFrac distance), whereas the columns indicate the different maternal lines (A, D, and G: maternal line 1; B, E, and H: maternal line 2; C, F, and I: maternal line 3). The *W* value and *P* value from the Mann-Whitney test are provided in the upper left corner.

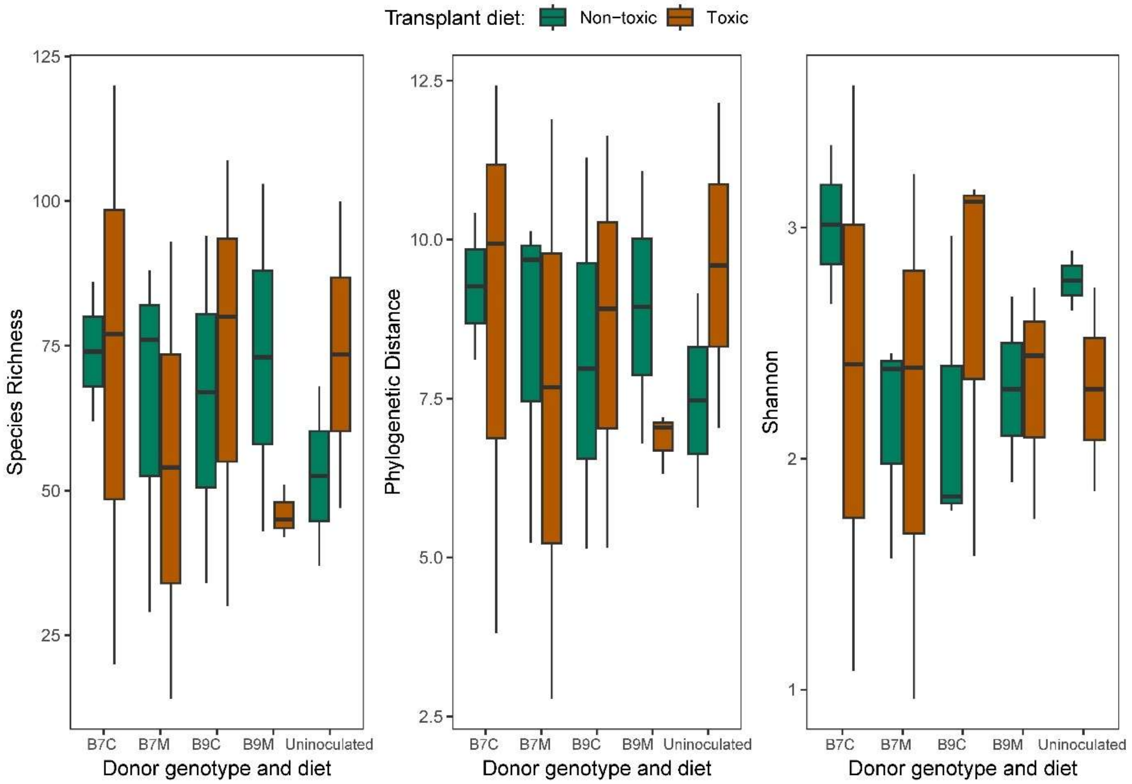

Figure S11: Boxplots of alpha diversity in the transplant phase. The alpha diversity metrics are given as separate panels (i.e., species richness, Faith's phylogenetic distance, and Shannon Index). The donor genotype and donor diet are shown on the x-axis whereas the colour indicates the transplant diet (green for non-toxic and orange for toxic).

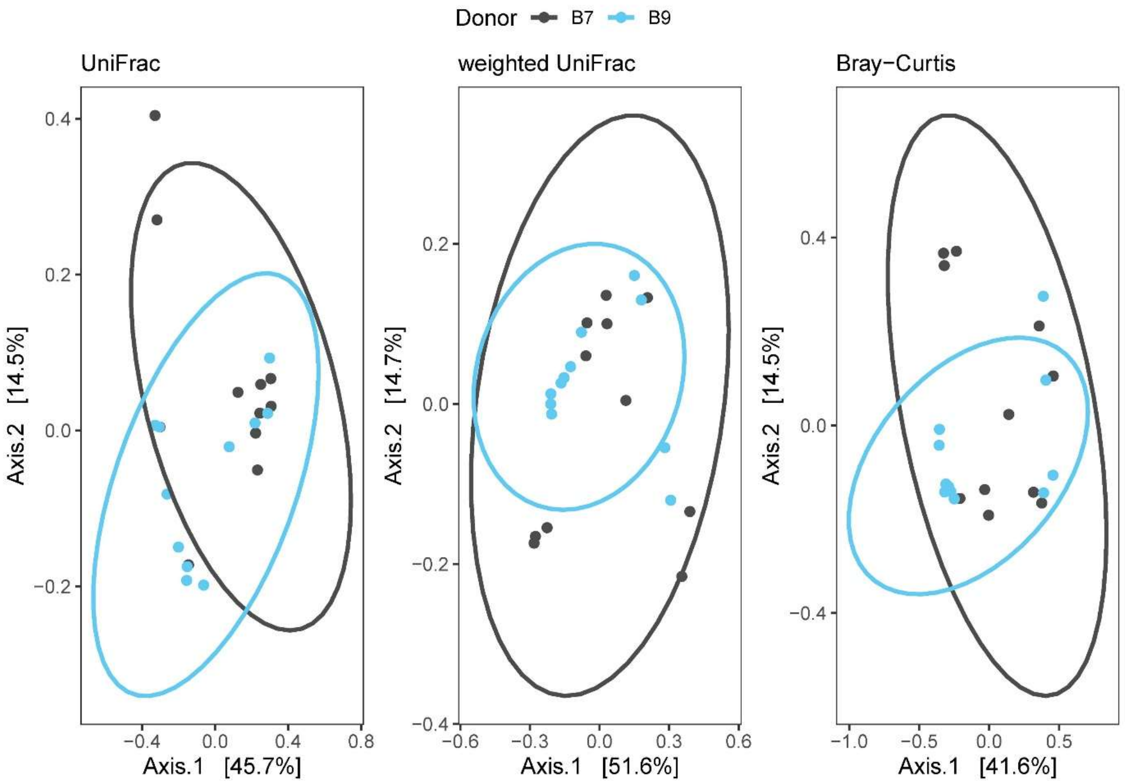

Figure S12: Beta diversity in transplants. When investigating the difference between donor clone and donor diet (and removing the treatment without microbial inoculum), the donor clone becomes significant in weighted UniFrac and marginally significant in UniFrac.

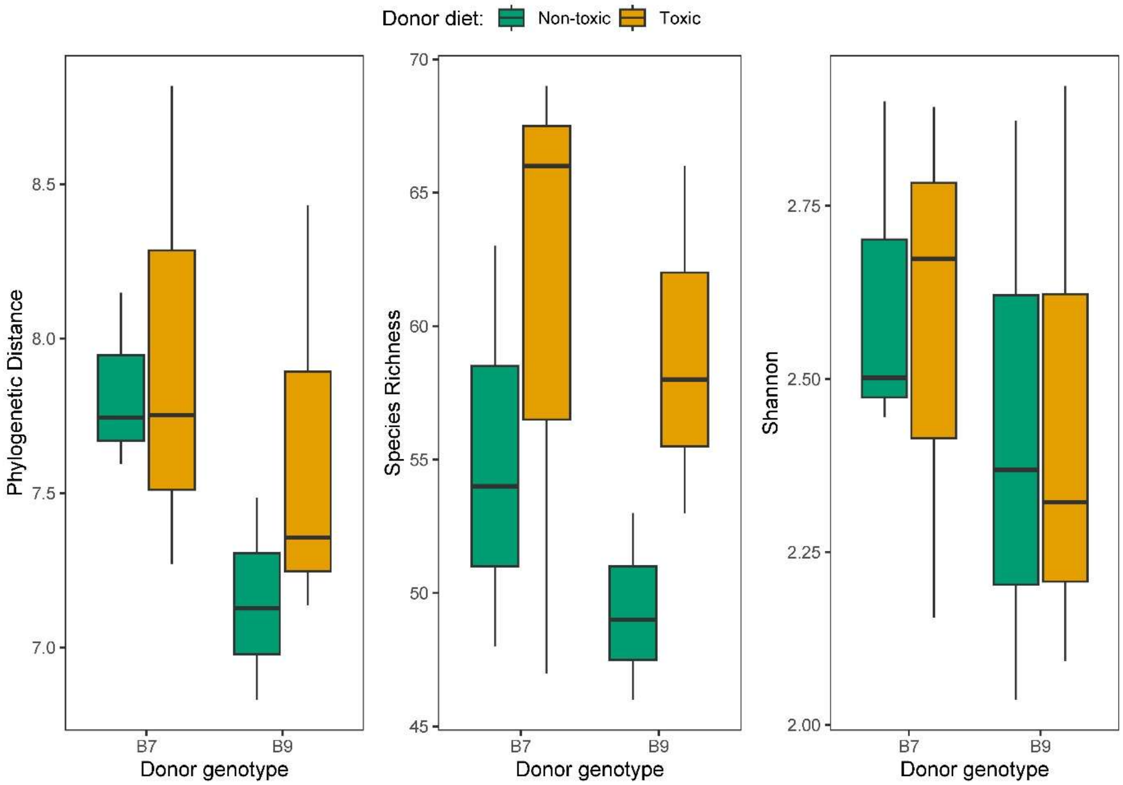

Figure S13: Boxplots of alpha diversity in the donor phase. The alpha diversity metrics are given as separate panels (i.e., Faith's phylogenetic distance, species richness, and Shannon Index). The donor genotypes are shown on the x-axis (i.e., B7 and B9) whereas the colour indicates the transplant diet (green for non-toxic and orange for toxic).

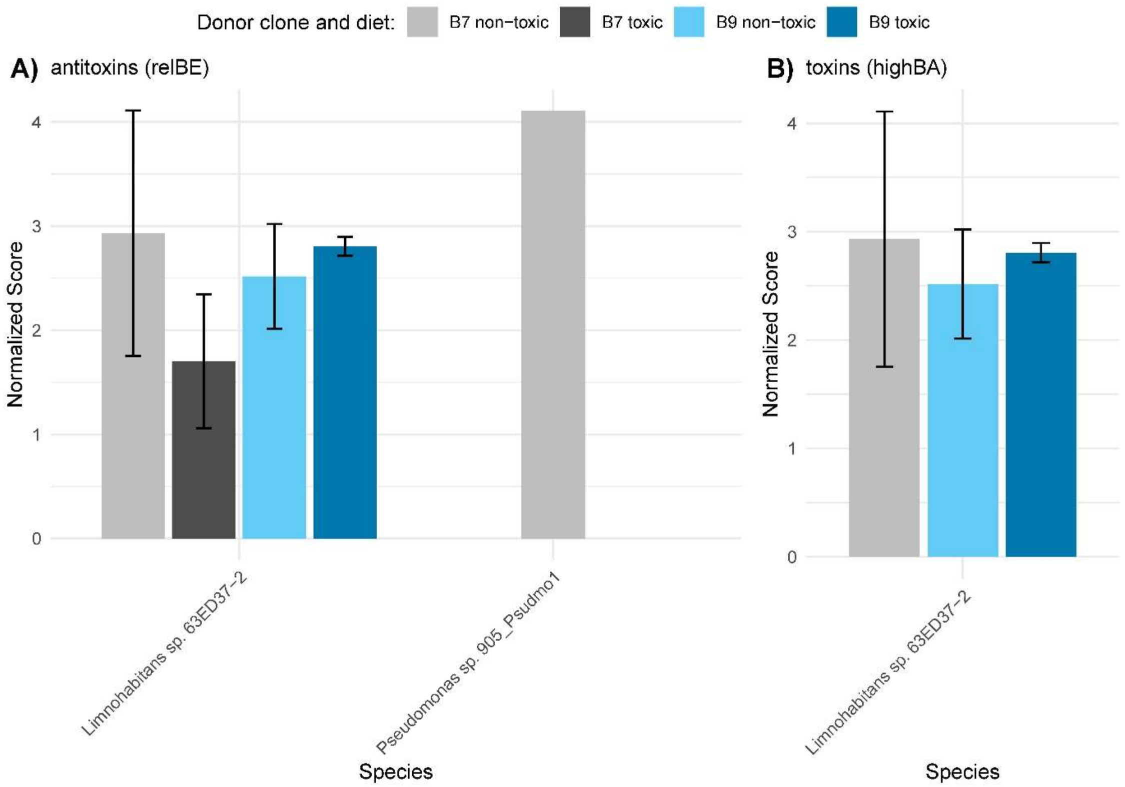

Figure S14: Toxins-antitoxins in the transplant phase. The toxin-antitoxin loci from non-host read assemblies were annotated using the TADB database. Filter thresholds are: ≥ 90% identity, E ≤ 1e–5, bit score ≥ 200, alignment ≥ 70% of the query, ≤ 5% mismatches, and ≤ 2 gap openings. The normalized scores are given for the (A) antitoxins and (B) toxics. The different treatments are shown in different colours with donor clone B7 in grey and donor clone B9 in blue, whereas non-toxic diets are brighter and toxic diets are darker. No matches were found for the uninoculated samples.

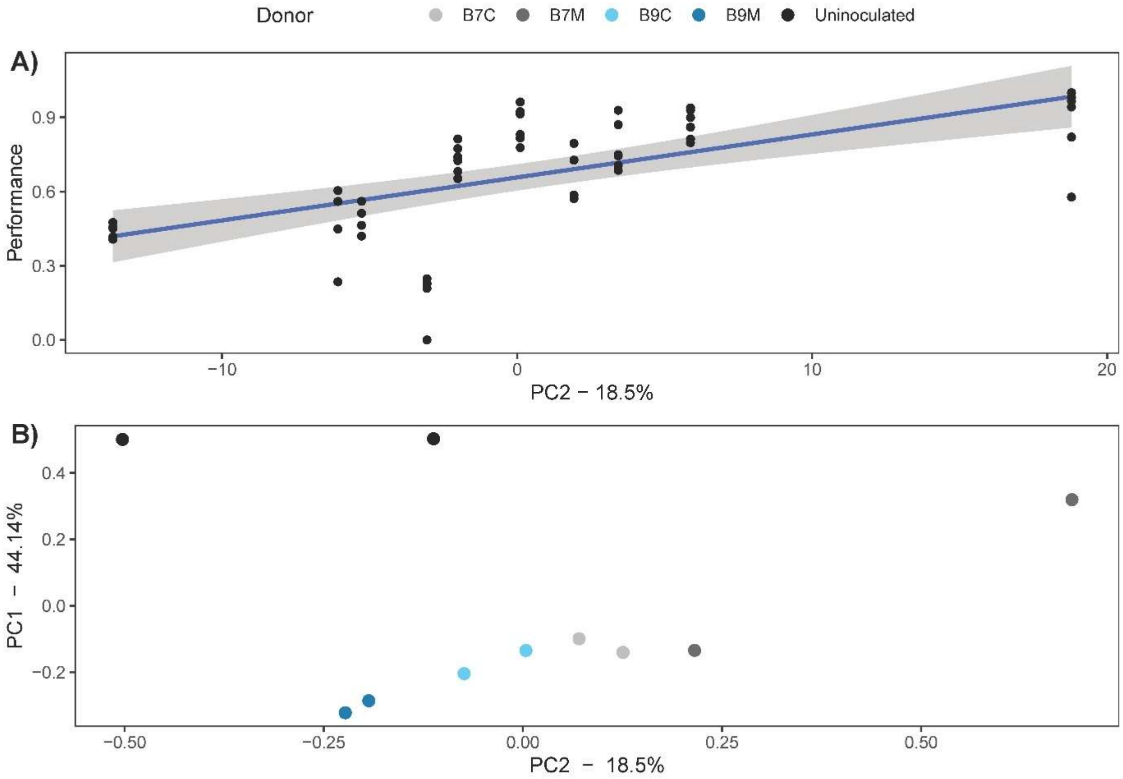

Figure S15: Functional profiling PCAs using abundance pathways from HUMAnN3 and comparing with host performance. (B) The PCA is generated using code adapted from a previously published method (Velsko et al., 2022). For each sample, the PCA reflects the abundance of microbial metabolic pathways. There were no pathways with PC loadings of ≥0.25. *Daphnia* host mapped reads are removed in this analysis. **(A)** The PC where host performance was significantly correlated with is presented.

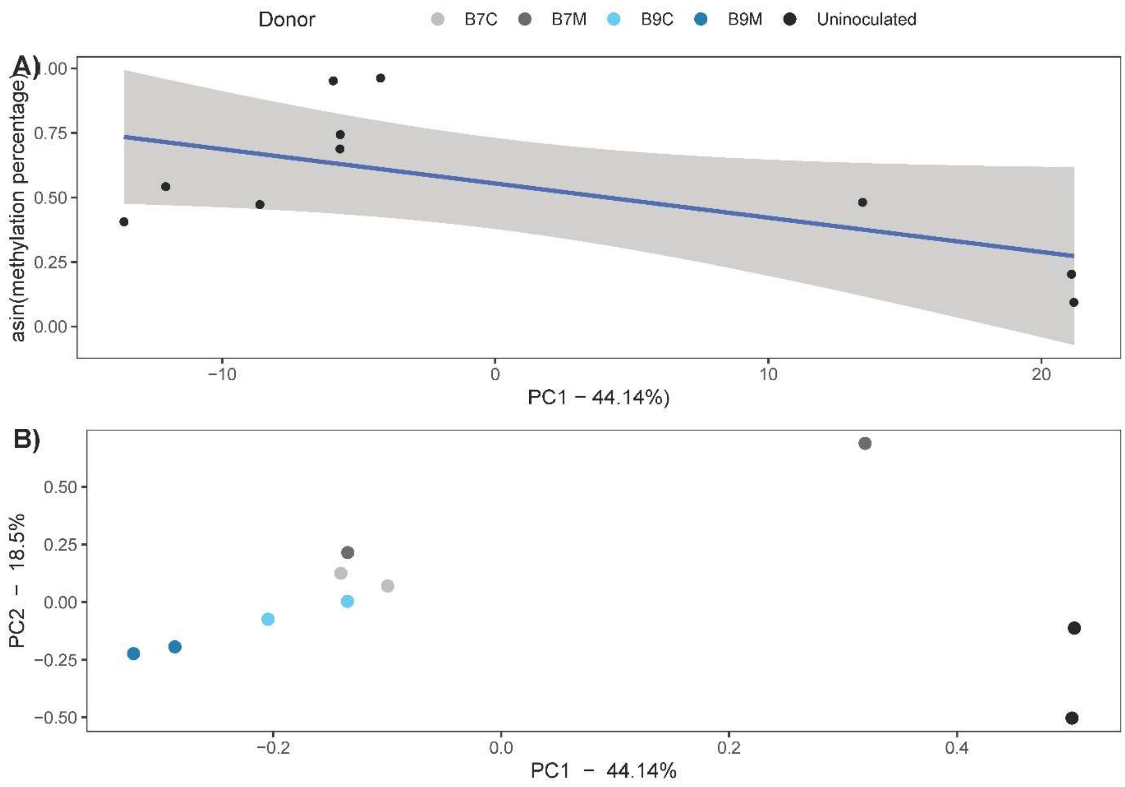

Figure S16: Functional profiling PCAs using abundance pathways from HUMAnN3 and comparing with DNA methylation level. (**B**) The PCA is generated using code adapted from a previously published method (Velsko et al., 2022). For each sample, the PCA reflects the abundance of microbial metabolic pathways. There were no pathways with PC loadings of ≥0.25. *Daphnia* host mapped reads are removed in this analysis. (**A**) The PC where DNA methylation levels were significantly correlated with is presented.

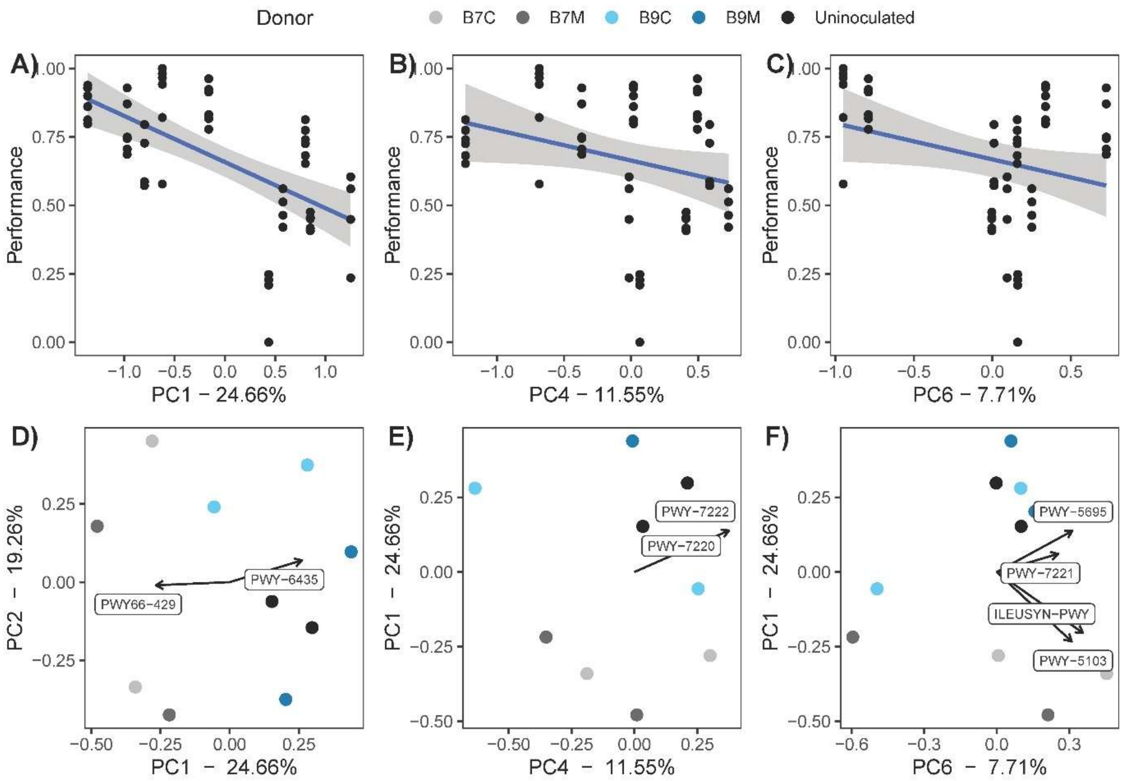

Figure S17: Functional profiling PCAs using coverage pathways from HUMAnN3 and comparing with host performance. (**D,E,F**) The PCAs are generated using code adapted from a previously published method (Velsko et al., 2022). For each sample, the PCA reflects variation among samples in both which pathways are present and how completely the pathways are covered (a threshold is used where only pathways that are covered for more than 50% are considered). Only pathways with PC loadings of ≥0.25 are visualised. *Daphnia* host mapped reads are removed in this analysis. (**A,B,C**) The PCs where host performance was significantly correlated with are presented. Combining both plots may indicate a potential positive correlation between host performance and PWY66-429 (fatty acid biosynthesis initiation), and a negative correlation with PWY-6435, PWY-7220, PWY-7222, PWY-5695, PWY-7221, PWY-5103, and ILEUSYN-PWY (more information on pathways in Table S12).

TABLES

Table S1: Assessment of the toxins of *M. aeruginosa* before and after UV treatment for the three different replicates.

	Before UV treatment	After UV treatment
Batch one	420 µg/L	352 µg/L
Batch two	488 µg/L	450 µg/L
Batch three	440 µg/L	444 µg/L

Table S2: mapping statistics from whole genome resequencing. The total number of reads, and the number of mapped and unmapped reads are given per sample, as well as the percentage of mapped and properly paired reads. In the donors and transplant diets, the C is the non-toxic diet (i.e., *Chlorella vulgaris*) and M toxic diet (i.e., *Microcystis aeruginosa*).

recipient	donor	Transplant diet	total	mapped	unmapped	mapped	properly paired
B9	B7C	C	12506561	11081763	1424798	88.6%	86.7%
B9	B7C	M	32081348	29912146	2169202	93.2%	91.7%
B9	B7M	C	16096208	15642420	453788	97.2%	95.5%
B9	B7M	M	18285621	17000783	1284838	93.0%	91.3%
B9	B9M	C	12560950	11076284	1484666	88.2%	86.7%
B9	B9M	M	11073628	10089151	984477	91.1%	89.7%
B9	B9C	C	13692822	12241321	1451501	89.4%	87.2%
B9	B9C	M	19155622	17754772	1400850	92.7%	91.0%
B9	uninoculated	C	13318474	13180350	138124	99.0%	97.3%
B9	uninoculated	M	15449358	15291801	157557	99.0%	97.5%

Table S3: summary statistics for the phenotypic measurements of the donor phase. A) The output of the fixed effects of the mixed effects coxme model. **B)** 1. The output of the Type II Wald χ^2 tests with body size as dependent variable for all time points. 2. The output of the Type II Wald χ^2 tests with body size as dependent variable for day 15. 3. The pairwise comparisons for the model with only day 15. **C)** The output of the Type II Wald χ^2 tests with total brood as dependent variable.

Donor phase					
**A) Survival**					
	**coef**	**exp(coef)**	**se(coef)**	***Z* value**	***P* value**
donor diet (toxic)	0.405	1.499	0.586	0.69	0.4890
donor clone (B9)	-0.031	0.970	0.633	-0.05	0.9610
donor diet (toxic) : donor clone (B9)	-1.326	0.266	1.021	-1.30	0.1940
**B) Body size**					
1. Type II Wald χ^2 tests: all time points					
	χ^2	**df**	***P* value**		
donor clone	0.179	1	0.6722		
donor diet	6.063	1	**0.0138**	*	
time	0.997	1	0.3181		
donor clone : donor diet	0.854	1	0.3554		
donor clone : time	3.743	1	0.0530	.	
donor diet : time	1.257	1	0.2621		
donor clone : donor diet : time	0.037	1	0.8484		
2. Type II Wald χ^2 tests: only at day 15					
	χ^2	**df**	***P* value**		
DonorClone	1.706	1	0.1915		
DonorDiet	27.535	1	**0.0000**	***	
DonorClone:DonorDiet	4.931	1	**0.0264**	*	
3. pairwise comparisons at day 15					
**donor clone: contrast**	**estimate**	**SE**	**df**	***t* ratio**	***P* value**
B7: non toxic - toxic	0.681	0.129	34	5.281	**<.0001**
B9: non toxic - toxic	0.276	0.129	34	2.140	**0.0396**
**donor diet: contrast**	**estimate**	**SE**	**df**	***t* ratio**	***P* value**
non toxic: B7 - B9	0.371	0.158	34	2.349	**0.0248**
toxic: B7 - B9	-0.034	0.158	34	-0.215	0.8309
**C) Total brood**					
Type II Wald χ^2 tests					
	χ^2	**df**	***P* value**		
donor clone	0.431	1	0.5115		
donor diet	11.208	1	0.0008	***	
donor clone : donor diet	2.906	1	0.0883	.	

Table S4: summary statistics for the phenotypic measurements of the transplant phase. A) 1. The output of the Type II Wald χ^2 tests with survival percentage as dependent variable. 2. The pairwise comparisons contrasting the donors (clone and diet). **B)** 1. The output of the Type II Wald χ^2 tests with body size as dependent variable. 2. The pairwise comparisons contrasting the donors (clone and diet), contrasting the donors per transplant diet (first non-toxic then toxic), and contrasting the transplant diets per donor (clone and diet).

Transplant phase					
**A) Survival percentage**					
1. Type II Wald χ^2 tests: all time points					
	**χ^2**	**df**	***P* value**		
donor (clone and diet)	31.459	4	**2.47E-06**	***	
transplant diet	0.654	1	0.4186		
donor : transplant diet	3.519	4	0.4750		
2. pairwise comparisons between donors (clone and diet)					
**donor: contrast**	**estimate**	**SE**	**df**	***t* ratio**	***P* value**
B7C-B7M	-0.071	0.084	18	-0.853	0.9102
B7C-B9C	-0.132	0.084	18	-1.577	0.5294
B7C-B9M	0.104	0.084	18	1.236	0.7312
B7C-no microbial inoculum	0.291	0.084	18	3.467	**0.0203**
B7M-B9C	-0.061	0.084	18	-0.725	0.9480
B7M-B9M	0.175	0.084	18	2.089	0.2670
B7M- no microbial inoculum	0.362	0.084	18	4.320	**0.0033**
B9C-B9M	0.236	0.084	18	2.813	0.0753
B9C- no microbial inoculum	0.423	0.084	18	5.044	**0.0007**
B9M- no microbial inoculum	0.187	0.084	18	2.231	0.2132
**B) Body size**					
1. Type II Wald χ^2 tests					
	**χ^2**	**df**	***P* value**		
donor (clone and diet)	92.860	4	**< 2e-16**	***	
transplant diet	0.129	1	0.7195		
donor : transplant diet	13.003	4	**0.0113**	*	
2. pairwise comparisons					
**contrast**	**estimate**	**SE**	**df**	***t* ratio**	***P* value**
**only contrasting the donors (all transplant diets)**					
B7C - B7M	-0.2968	0.0611	121	-4.862	**<.0001**
B7C - B9C	-0.3695	0.06	121	-6.155	**<.0001**
B7C - B9M	-0.2155	0.0623	121	-3.459	**0.0066**
B7C - no microbial inoculum	0.1412	0.0642	121	2.2	0.1866
B7M - B9C	-0.0726	0.0589	121	-1.233	0.7320
B7M - B9M	0.0813	0.0613	121	1.327	0.6749
B7M - no microbial inoculum	0.4381	0.0631	121	6.944	**<.0001**
B9C - B9M	0.154	0.0602	121	2.556	0.0853
B9C - no microbial inoculum	0.5107	0.0621	121	8.218	**<.0001**
B9M - no microbial inoculum	0.3567	0.0643	121	5.544	**<.0001**
**transplant diet: non-toxic**					
B7C - B7M	-0.451	0.086	121	-5.224	**<.0001**
B7C - B9C	-0.323	0.085	121	-3.795	**0.0021**

B7C - B9M	-0.268	0.086	121	-3.102	**0.0199**
B7C - no microbial inoculum	0.128	0.092	121	1.395	0.6323
B7M - B9C	0.129	0.083	121	1.549	0.5332
B7M - B9M	0.184	0.085	121	2.165	0.2003
B7M - no microbial inoculum	0.580	0.090	121	6.422	**<.0001**
B9C - B9M	0.055	0.083	121	0.657	0.9649
B9C - no microbial inoculum	0.451	0.089	121	5.067	**<.0001**
B9M - no microbial inoculum	0.396	0.091	121	4.373	**0.0002**
**transplant diet: toxic**					
B7C - B7M	-0.142	0.086	121	-1.648	0.4699
B7C - B9C	-0.416	0.085	121	-4.904	**<.0001**
B7C - B9M	-0.163	0.090	121	-1.817	0.3686
B7C - no microbial inoculum	0.154	0.090	121	1.715	0.4284
B7M - B9C	-0.274	0.083	121	-3.293	**0.0112**
B7M - B9M	-0.021	0.088	121	-0.239	0.9993
B7M - no microbial inoculum	0.297	0.088	121	3.361	**0.0090**
B9C - B9M	0.253	0.087	121	2.915	**0.0338**
B9C - no microbial inoculum	0.571	0.087	121	6.572	**<.0001**
B9M - no microbial inoculum	0.318	0.091	121	3.472	**0.0063**
**donor: B7C**					
non-toxic-toxic	-0.0827	0.0881	121	-0.939	0.3496
**donor: B7M**					
non-toxic-toxic	0.2264	0.0847	121	2.674	**0.0085**
**donor: B9C**					
non-toxic-toxic	-0.1767	0.0818	121	-2.160	**0.0328**
**donor: B9M**					
non-toxic-toxic	0.0216	0.0882	121	0.245	0.8066
**donor: no microbial inoculum**					
non-toxic-toxic	-0.0568	0.0936	121	-0.606	0.5453

Table S5: summary statistics for exploring the microbiota-reducing treatment in the recipient phase using the alpha diversity. A) The output of the Type II Wald χ^2 tests for Faith's phylogenetic distance. **B)** The pairwise comparisons between microbiota-reducing and control treatments per recipient clone for Faith's phylogenetic distance. **C)** The output of the Type II Wald χ^2 tests for species richness. **D)** The pairwise comparisons between microbiota-reducing and control treatments per recipient clone for species richness. **E)** The output of the Type II Wald χ^2 tests for Shannon Index. **F)** The pairwise comparisons between microbiota-reducing and control treatments per recipient clone for Shannon Index.

Recipient phase					
**A) alpha diversity: Faith's phylogenetic distance**					
1. Type II Wald χ^2 tests					
	**χ^2**	**df**	***P* value**		
Recipient	0.003	1	0.9554		
Microbiota-reduced	4.417	1	**0.0356**	*	
Recipient:Microbiota-reduced	7.124	1	**0.0076**	**	
2. pairwise comparisons between microbiota-reducing and control treatments per recipient clone					
**donor: contrast**	**estimate**	**SE**	**df**	***t* ratio**	***P* value**
B7: untreated - treated	-0.4	0.996	7	-0.401	0.7003
B9: untreated - treated	3.36	0.996	7	3.373	0.0119
**B) alpha diversity: species richness**					
1. Type II Wald χ^2 tests					
	**χ^2**	**df**	***P* value**		
Recipient	0.085	1	0.7701		
Microbiota-reduced	3.063	1	**0.0801**	.	
Recipient:Microbiota-reduced	9.370	1	**0.0022**	**	
2. pairwise comparisons between microbiota-reducing and control treatments per recipient clone					
**donor: contrast**	**estimate**	**SE**	**df**	***t* ratio**	***P* value**
B7: untreated - treated	-0.158	0.202	Inf	-0.781	0.4347
B9: untreated - treated	0.755	0.22	Inf	3.437	0.0006
**C) alpha diversity: Shannon Index**					
1. Type II Wald χ^2 tests					
	**χ^2**	**df**	***P* value**		
Recipient	3.068	1	0.0799	.	
Microbiota-reduced	3.071	1	0.0797	.	
Recipient:Microbiota-reduced	9.171	1	**0.0025**	**	
2. pairwise comparisons between microbiota-reducing and control treatments per recipient clone					
**donor: contrast**	**estimate**	**SE**	**df**	***t* ratio**	***P* value**
B7: untreated - treated	-0.357	0.396	7	-0.902	0.3969
B9: untreated - treated	1.338	0.396	7	3.38	0.0118

Table S6: summary statistics for exploring the microbiota-reducing and control treatments in the recipient phase using the beta diversity. A) Results for the weighted UniFrac with the results of the homogeneity of multivariate dispersions (1) and of the PERMANOVA test (2). **B)** Results for the unweighted UniFrac with the results of the homogeneity of multivariate dispersions (1) and of the PERMANOVA test (2). **C)** Results for the Bray-Curtis dissimilarity with the results of the homogeneity of multivariate dispersions (1) and of the PERMANOVA test (2).

Recipient phase						
**A) weighted UniFrac**						
1. Homogeneity of multivariate dispersions						
	**df**	**Sum Sq.**	**Mean Sq.**	***F* value**	**Perm.**	***P* value**
**Groups: microbiota-reducing**	1	0.00804	0.008042	0.8	999	0.374
Residuals	10	0.09592	0.0096			
**Groups: recipient clone**	1	0.01854	0.0185	1.2	999	0.318
Residuals	10	0.14953	0.0150			
2. PERMANOVA						
	**df**	**SumOfSqs**	***R^2***	***F* value**	***P* value**	
microbiota-reducing	1	0.1988	0.2522	5.4	**0.01299**	*
recipient clone	1	0.1662	0.2108	4.5	**0.02897**	*
microbiota-reducing: recipient clone	1	0.1307	0.1659	3.6	0.05894	.
Residual	8	0.2925	0.3711			
Total	11	0.7881	1			
**B) unweighted UniFrac**						
1. Homogeneity of multivariate dispersions						
	**df**	**Sum Sq.**	**Mean Sq.**	***F* value**	**Perm.**	***P* value**
**Groups: microbiota-reducing**	1	0.000136	0.000136	0.01	999	0.852
Residuals	10	0.13185	0.013185			
**Groups: recipient clone**	1	0.006486	0.006486	0.4	999	0.53
Residuals	10	0.149649	0.014965			
2. PERMANOVA						
	**df**	**SumOfSqs**	***R^2***	***F* value**	***P* value**	
microbiota-reducing	1	0.1956	0.1761	2.2	**0.003996**	**
recipient clone	1	0.0698	0.0628	0.8	0.63037	
microbiota-reducing: recipient clone	1	0.1420	0.1278	1.6	**0.035964**	*
Residual	8	0.7035	0.6333			
Total	11	1.1109	1			
**C) Bray-Curtis**						
1. Homogeneity of multivariate dispersions						
	**df**	**Sum Sq.**	**Mean Sq.**	***F* value**	**Perm.**	***P* value**
**Groups: microbiota-reducing**	1	0.00133	0.001334	0.04	999	0.842
Residuals	10	0.30497	0.0305			
**Groups: recipient clone**	1	0.02449	0.0245	0.4	999	0.532
Residuals	10	0.55155	0.0552			
2. PERMANOVA						
	**df**	**SumOfSqs**	***R^2***	***F* value**	***P* value**	
microbiota-reducing	1	0.6095	0.2674	5.7	**0.002997**	**
recipient clone	1	0.4010	0.1759	3.7	**0.036963**	*
microbiota-reducing: recipient clone	1	0.4118	0.1806	3.8	**0.027972**	*

Residual	8	0.8574	0.3761
Total	11	2.2796	1

Table S7: summary statistics for donor fidelity (the transplant phase using beta diversity). A) Results for the weighted UniFrac with the results of the homogeneity of multivariate dispersions (1) and of the PERMANOVA test (2). B) Results for the unweighted UniFrac with the results of the homogeneity of multivariate dispersions (1) and of the PERMANOVA test (2). C) Results for the Bray-Curtis dissimilarity with the results of the homogeneity of multivariate dispersions (1) and of the PERMANOVA test (2). D) Results of the post hoc testing for the interaction found using Bray-Curtis dissimilarity. The microbial inoculum had a marginal significance effect on the Bray-Curtis dissimilarity in the third maternal line.

Recipient phase							
**A) weighted UniFrac**							
1. Homogeneity of multivariate dispersions							
	**df**	**Sum Sq.**	**Mean Sq.**	***F* value**	**Perm.**	***P* value**	
**Groups: microbial inoculum**	3	0.0178	0.0059	0.3	999	0.874	
Residuals	18	0.4230	0.0235				
**Groups: maternal line**	2	0.0217	0.0108	1.8	999	0.2	
Residuals	19	0.1142	0.0060				
2. PERMANOVA							
	**df**	**SumOfSqs**	**R^2**	***F* value**	***P* value**		
inoculum	3	0.1871	0.0987	1.5	0.157		
maternal line	2	0.8990	0.4741	10.6	**0.001**	***	
inoculum:maternal line	6	0.3876	0.2044	1.5	0.127		
Residual	10	0.4225	0.2228				
Total	21	1.8962	1.0000				
**B) unweighted UniFrac**							
1. Homogeneity of multivariate dispersions							
	**df**	**Sum Sq.**	**Mean Sq.**	***F* value**	**Perm.**	***P* value**	
**Groups: microbial inoculum**	3	0.0245	0.0082	0.5	999	0.677	
Residuals	18	0.2984	0.0166				
**Groups: maternal line**	2	0.0101	0.0051	0.9	999	0.458	
Residuals	19	0.1093	0.0058				
2. PERMANOVA							
	**df**	**SumOfSqs**	**R^2**	***F* value**	***P* value**		
inoculum	3	0.3241	0.1112	1.4	0.187		
maternal line	2	1.0928	0.3749	6.9	0.085	.	
inoculum:maternal line	6	0.7022	0.2409	1.5	0.103		
Residual	10	0.7957	0.2730				
Total	21	2.9149	1.0000				
**C) Bray-Curtis**							
1. Homogeneity of multivariate dispersions							
	**df**	**Sum Sq.**	**Mean Sq.**	***F* value**	**Perm.**	***P* value**	
**Groups: microbial inoculum**	3	0.0180	0.0060	0.1	999	0.943	
Residuals	18	0.8135	0.0452				
**Groups: maternal line**	2	0.0192	0.0096	1.2	999	0.321	
Residuals	19	0.1484	0.0078				
2. PERMANOVA							
	**df**	**SumOfSqs**	**R^2**	***F* value**	***P* value**		
inoculum	3	0.6098	0.1125	1.7	0.087	.	

maternal line	2	2.1045	0.3882	8.7	**0.009**	**
inoculum:maternal line	6	1.4993	0.2766	2.1	**0.016**	*
Residual	10	1.2072	0.2227			
Total	21	5.4208	1.0000			

D) Bray-Curtis (PERMANOVA with inoculum per maternal line)

	**df**	**SumOfSqs**	R^2	***F* value**	***P* value**	
Maternal line 1						
Model	3	0.73575	0.69585	1.5	0.1111	
Residual	2	0.32159	0.30415			
Total	5	1.05734	1			
Maternal line 2						
Model	3	0.82054	0.61261	2.1	0.223	
Residual	4	0.51888	0.38739			
Total	7	1.33942	1			
Maternal line 3						
Model	3	0.62603	0.63061	2.3	0.060	.
Residual	4	0.36671	0.36939			
Total	7	0.99274	1			

Table S8: summary statistics for the transplant phase using the alpha diversity. A) The output of the Type II Wald χ^2 tests for Faith's phylogenetic distance with all treatments (1) and excluding the treatment without microbial inoculum to further investigate donor clone and donor diet (2). **B)** The output of the Type II Wald χ^2 tests for Shannon Index with all treatments (1) and excluding the treatment without microbial inoculum to further investigate donor clone and donor diet (2). **C)** The output of the Type II Wald χ^2 tests for species richness. **C)** The output of the Type II Wald χ^2 tests for species richness with all treatments (1), the pairwise comparisons between donors for the non-toxic transplant diet (2), the pairwise comparisons between donors for the toxic transplant diet (3), the pairwise comparisons between transplant diets per donor (4), and the Type II Wald χ^2 test excluding the treatment without microbial inoculum to further investigate donor clone and donor diet (5).

Transplant phase			
**A) alpha diversity: Faith's phylogenetic distance**			
1. Type II Wald χ^2 tests			
	**χ^2**	**df**	***P* value**
donor (clone and diet)	4.6277	4	0.328
transplant diet	1.7462	1	0.186
donor :transplant diet	7.4585	4	0.114
2. Type II Wald χ^2 tests (excluding the treatment without microbial inoculum)			
	**χ^2**	**df**	***P* value**
donor diet	1.6698	1	0.196
donor clone	0.2469	1	0.619
transplant diet	2.8176	1	0.093 .
donor diet : donor clone	1.0151	1	0.314
donor diet : transplant diet	1.1693	1	0.280
donor clone: transplant diet	0.0062	1	0.937
donor diet : donor clone : transplant diet	2.3595	1	0.125
**B) alpha diversity: Shannon Index**			
1. Type II Wald χ^2 tests			
	**χ^2**	**df**	***P* value**
donor (clone and diet)	4.6833	4	0.321
transplant diet	0.1578	1	0.691
donor :transplant diet	4.7006	4	0.319
2. Type II Wald χ^2 tests (excluding the treatment without microbial inoculum)			
	**χ^2**	**df**	***P* value**
donor diet	2.2522	1	0.133
donor clone	0.0497	1	0.824
transplant diet	0.0190	1	0.890
donor diet : donor clone	0.9644	1	0.326
donor diet : transplant diet	0.1205	1	0.729
donor clone: transplant diet	1.5598	1	0.212
donor diet : donor clone : transplant diet	2.2828	1	0.131
**C) alpha diversity: species richness**			
1. Type II Wald χ^2 tests			
	**χ^2**	**df**	***P* value**
donor (clone and diet)	4.4805	4	0.345
transplant diet	2.2259	1	0.136

donor :transplant diet	9.7995	4	**0.044**	*	
2. pairwise comparisons between donors (non-toxic transplant diet)					
**contrast: donors**	**estimate**	**SE**	**df**	***z* ratio**	***P* value**
B7C - B7M	0.2983	0.216	Inf	1.380	0.641
B7C - B9C	0.2886	0.215	Inf	1.341	0.665
B7C - B9M	0.0002	0.225	Inf	0.001	1.000
B7C - no microbial inoculum	0.3226	0.245	Inf	1.318	0.680
B7M - B9C	-0.0097	0.194	Inf	-0.050	1.000
B7M - B9M	-0.2981	0.216	Inf	-1.383	0.639
B7M - no microbial inoculum	0.0243	0.236	Inf	0.103	1.000
B9C - B9M	-0.2884	0.215	Inf	-1.343	0.664
B9C - no microbial inoculum	0.0340	0.235	Inf	0.144	1.000
B9M - no microbial inoculum	0.3224	0.244	Inf	1.318	0.680
3. pairwise comparisons between donors (toxic transplant diet)					
**contrast: donors**	**estimate**	**SE**	**df**	***z* ratio**	***P* value**
B7C - B7M	0.2807	0.205	Inf	1.367	0.649
B7C - B9C	-0.0454	0.188	Inf	-0.242	0.999
B7C - B9M	0.4584	0.217	Inf	2.117	0.213
B7C - no microbial inoculum	-0.1394	0.210	Inf	-0.664	0.964
B7M - B9C	-0.3262	0.202	Inf	-1.614	0.489
B7M - B9M	0.1777	0.230	Inf	0.774	0.938
B7M - no microbial inoculum	-0.4201	0.226	Inf	-1.858	0.340
B9C - B9M	0.5038	0.214	Inf	2.357	0.127
B9C - no microbial inoculum	-0.0939	0.210	Inf	-0.448	0.992
B9M - no microbial inoculum	-0.5977	0.235	Inf	-2.548	0.080
4. pairwise comparisons between transplant diets per donor					
**contrast**	**estimate**	**SE**	**df**	***z* ratio**	***P* value**
B7C					
non-toxic - toxic	0.233	0.217	Inf	1.076	0.282
B7M					
non-toxic - toxic	0.215	0.207	Inf	1.041	0.298
B9C					
non-toxic - toxic	-0.101	0.189	Inf	-0.533	0.594
B9M					
non-toxic - toxic	0.691	0.239	Inf	2.894	0.004
no microbial inoculum					
non-toxic - toxic	-0.229	0.259	Inf	-0.884	0.377
5. Type II Wald χ^2 tests (excluding treatment without microbial inoculum)					
	**χ^2**	**df**	***P* value**		
donor diet	3.0463	1	0.081	.	
donor clone	0.0313	1	0.860		
transplant diet	3.3930	1	0.065	.	
donor diet : donor clone	0.4958	1	0.481		
donor diet : transplant diet	2.8675	1	0.090	.	
donor clone: transplant diet	0.0222	1	0.882		
donor diet : donor clone : transplant diet	3.0147	1	0.083	.	

Table S9: summary statistics for the transplant phase using beta diversity. A) Results for the weighted UniFrac with the results of the homogeneity of multivariate dispersions (1) and of the PERMANOVA test (2). **B)** Results for the unweighted UniFrac with the results of the homogeneity of multivariate dispersions (1) and of the PERMANOVA test (2). **C)** Results for the Bray-Curtis dissimilarity with the results of the homogeneity of multivariate dispersions (1) and of the PERMANOVA test (2).

Transplant phase

**A) weighted UniFrac**						
1. Homogeneity of multivariate dispersions						
	**df**	**Sum Sq.**	**Mean Sq.**	***F* value**	**Perm.**	***P* value**
**Groups: donor clone**	1	0.0181	0.0181	0.87	999	0.348
Residuals	20	0.4163	0.0208			
**Groups: donor diet**	1	0.0007	0.0007	0.04	999	0.871
Residuals	20	0.3349	0.0167			
**Groups: transplant diet**	1	0.0003	0.0003	0.02	999	0.900
Residuals	20	0.3299	0.0165			
2. PERMANOVA						
	**df**	**SumOfSqs**	R^2	***F* value**	***P* value**	
donor clone	1	0.1127	0.0594	1.0	**0.048**	*
donor diet	1	0.0415	0.0219	0.4	0.688	
transplant diet	1	0.0292	0.0154	0.3	0.893	
donor clone : donor diet	1	0.0376	0.0199	0.3	0.799	
donor clone : transplant diet	1	0.0324	0.0171	0.3	0.805	
donor diet : transplant diet	1	0.0270	0.0142	0.2	0.859	
donor clone : donor diet : transplant diet	1	0.1018	0.0537	0.9	0.222	
Residual	14	1.5140	0.7984			
Total	21	1.8962	1.0000			
**B) unweighted UniFrac**						
1. Homogeneity of multivariate dispersions						
	**df**	**Sum Sq.**	**Mean Sq.**	***F* value**	**Perm.**	***P* value**
**Groups: donor clone**	1	0.0200	0.0200	1.5	999	0.202
Residuals	20	0.2693	0.0135			
**Groups: donor diet**	1	0.0008	0.0008	0.1	999	0.721
Residuals	20	0.1055	0.0053			
**Groups: transplant diet**	1	0.0030	0.0030	0.6	999	0.445
Residuals	20	0.0925	0.0046			
2. PERMANOVA						
	**df**	**SumOfSqs**	R^2	***F* value**	***P* value**	
donor clone	1	0.1980	0.0679	1.2	**0.027**	*
donor diet	1	0.0578	0.0198	0.4	0.862	
transplant diet	1	0.0753	0.0258	0.5	0.726	
donor clone : donor diet	1	0.0697	0.0239	0.4	0.782	
donor clone : transplant diet	1	0.1015	0.0348	0.6	0.395	
donor diet : transplant diet	1	0.0884	0.0303	0.5	0.516	
donor clone : donor diet : transplant diet	1	0.0512	0.0176	0.3	0.933	
Residual	14	2.2731	0.7798			
Total	21	2.9149	1.0000			

C) Bray-Curtis

1. Homogeneity of multivariate dispersions						
	**df**	**Sum Sq.**	**Mean Sq.**	***F* value**	**Perm.**	***P* value**
**Groups: donor clone**	1	0.0024	0.0024	0.061	999	0.795
Residuals	20	0.7812	0.0391			
**Groups: donor diet**	1	0.0123	0.0123	0.664	999	0.440
Residuals	20	0.3693	0.0185			
**Groups: transplant diet**	1	0.0002	0.0001	0.008	999	0.923
Residuals	20	0.3662	0.0183			
2. PERMANOVA						
	**df**	**SumOfSqs**	R^2	***F* value**	***P* value**	
donor clone	1	0.2993	0.0552	1.0	0.108	
donor diet	1	0.1848	0.0341	0.6	0.404	
transplant diet	1	0.0604	0.0112	0.2	0.996	
donor clone : donor diet	1	0.1272	0.0235	0.4	0.826	
donor clone : transplant diet	1	0.1521	0.0281	0.5	0.584	
donor diet : transplant diet	1	0.1698	0.0313	0.6	0.478	
donor clone : donor diet : transplant diet	1	0.2038	0.0376	0.7	0.475	
Residual	14	4.2233	0.7791			
Total	21	5.4208	1.0000			

Table S10: summary statistics donor phase using the alpha diversity. A) The output of the Type II Wald χ^2 tests for Faith's phylogenetic distance. **B)** The output of the Type II Wald χ^2 tests for species richness. **C)** The output of the Type II Wald χ^2 tests for Shannon Index.

Donor phase				
**A) alpha diversity: Faith's phylogenetic distance**				
1. Type II Wald χ^2 tests				
	χ^2	**df**	***P* value**	
donor clone	3.384	1	0.06583	.
donor diet	1.3015	1	0.25393	
donor clone : donor diet	0.4887	1	0.4845	
**B) alpha diversity: species richness**				
1. Type II Wald χ^2 tests				
	χ^2	**df**	***P* value**	
donor clone	0.7191	1	0.39645	
donor diet	3.14	1	0.07639	.
donor clone : donor diet	0.273	1	0.60134	
**C) alpha diversity: Shannon Index**				
1. Type II Wald χ^2 tests				
	χ^2	**df**	***P* value**	
donor clone	0.8011	1	0.3708	
donor diet	0.004	1	0.9497	
donor clone : donor diet	0.0309	1	0.8604	

Table S11: summary statistics for the donor phase using beta diversity. A) Results for the weighted UniFrac with the results of the homogeneity of multivariate dispersions (1) and of the PERMANOVA test (2). **B)** Results for the unweighted UniFrac with the results of the homogeneity of multivariate dispersions (1) and of the PERMANOVA test (2). **C)** Results for the Bray-Curtis dissimilarity with the results of the homogeneity of multivariate dispersions (1) and of the PERMANOVA test (2).

Recipient phase						
**A) weighted UniFrac**						
1. Homogeneity of multivariate dispersions						
	**df**	**Sum Sq.**	**Mean Sq.**	***F* value**	**Perm.**	***P* value**
**Groups: donor clone**	1	0.00001	6.8E-06	0.0004	999	0.991
Residuals	10	0.18264	0.0183			
**Groups: donor diet**	1	0.00074	0.0007	0.0456	999	0.832
Residuals	10	0.16279	0.0163			
2. PERMANOVA						
	**df**	**SumOfSqs**	R^2	***F* value**	***P* value**	
donor clone	1	0.0398	0.0692	0.7	0.527	
donor diet	1	0.0259	0.0451	0.4	0.741	
donor clone : donor diet	1	0.0241	0.0419	0.4	0.756	
Residual	8	0.4848	0.8439			
Total	11	0.5745	1			
**B) unweighted UniFrac**						
1. Homogeneity of multivariate dispersions						
	**df**	**Sum Sq.**	**Mean Sq.**	***F* value**	**Perm.**	***P* value**
**Groups: donor clone**	1	0.000165	0.000165	0.022	999	0.885
Residuals	10	0.074637	0.007464			
**Groups: donor diet**	1	0.000065	6.48E-05	0.009	999	0.937
Residuals	10	0.070849	0.007085			
2. PERMANOVA						
	**df**	**SumOfSqs**	R^2	***F* value**	***P* value**	
donor clone	1	0.0401	0.0533	0.5	0.8581	
donor diet	1	0.0643	0.0854	0.9	0.2368	
donor clone : donor diet	1	0.0553	0.0735	0.7	0.5325	
Residual	8	0.5927	0.7878			
Total	11	0.7523	1			
**C) Bray-Curtis**						
1. Homogeneity of multivariate dispersions						
	**df**	**Sum Sq.**	**Mean Sq.**	***F* value**	**Perm.**	***P* value**
**Groups: donor clone**	1	0.00169	0.001687	0.05	999	0.832
Residuals	10	0.32658	0.0327			
**Groups: donor diet**	1	0.00079	0.0008	0.04	999	0.84
Residuals	10	0.20741	0.0207			
2. PERMANOVA						
	**df**	**SumOfSqs**	R^2	***F* value**	***P* value**	
donor clone	1	0.0981	0.0702	0.7	0.2967	
donor diet	1	0.0703	0.0503	0.5	0.5594	
donor clone : donor diet	1	0.0607	0.0434	0.4	0.6933	

Residual	8	1.1688	0.8361
Total	11	1.3978	1

Table S12: overview of the important pathways explaining performance and DNA methylation. A) Functional pathways correlated to performance. No pathways were well explaining the PCs for abundance. **B)** Functional pathways correlated to DNA methylation.

**A) Performance - coverage**			
**positive**			
PWY66-429	fatty acid biosynthesis initiation (mitochondria)	Initiates mitochondrial fatty acid synthesis by using acetyl-CoA to build short acyl chains that serve as precursors for lipoic acid and other cofactors.	Asselman et al. (2017) found that fatty acid-related gene sets were associated with reproductive output in *Daphnia* under pollutant stress.
**negative**			
PWY-6435	4-hydroxybenzoate biosynthesis III (plants)	Produces 4-hydroxybenzoate, a precursor for ubiquinone (coenzyme Q) biosynthesis.	Pathway originally described in plants but also present in some bacteria.
PWY-7220	adenosine nucleotides de novo biosynthesis II	Synthesizes adenosine nucleotides de novo, essential for DNA/RNA synthesis.	
PWY-7222	guanosine nucleotides de novo biosynthesis II	Synthesizes guanosine nucleotides de novo, essential for DNA/RNA synthesis.	
PWY-5695	inosine 5'-phosphate degradation	Breaks down IMP, a purine nucleotide, into hypoxanthine and other metabolites.	
PWY-7221	guanosine ribonucleotides de novo biosynthesis	Synthesizes guanosine ribonucleotides from scratch, essential for DNA/RNA synthesis.	
PWY-5103	L-isoleucine biosynthesis III	Synthesizes the essential amino acid isoleucine, important for protein synthesis and energy.	
ILEUSYN-PWY	L-isoleucine biosynthesis I (from threonine)	Synthesizes the essential amino acid isoleucine from threonine.	
**B) DNA methylation - coverage**			
**positive**			
PWY66-429	fatty acid biosynthesis initiation (mitochondria)	Initiates mitochondrial fatty acid synthesis, producing precursors like acetyl-CoA for energy metabolism.	Fatty acid metabolism has been linked to epigenetic regulation via acetyl-CoA availability, which can influence histone acetylation and methylation.
**negative**			
PWY-6435	4-hydroxybenzoate biosynthesis III (plants)	Produces 4-hydroxybenzoate, a precursor for ubiquinone (coenzyme Q) in plants.	

Table S13: summary statistics for the DNA methylation percentage (arcsine square root transformed) of the transplant phase. A) The output of the Type II Wald χ^2 tests for the general model. **B)** The output of the Type II Wald χ^2 tests for non-toxic transplant diet. **C)** The pairwise comparisons for the non-toxic transplant diet contrasting the donor clones. **D)** The output of the Type II Wald χ^2 tests for toxic transplant diet. **E)** The pairwise comparisons for the toxic transplant diet contrasting the donor clones. **F)** The output of the Type II Wald χ^2 test excluding the treatment without microbial inoculum to investigate donor clone and donor diet separately.

Transplant phase					
**DNA methylation percentage (arcsine square root transformation)**					
A. Type II Wald χ^2 tests: general					
	χ^2	**df**	***P* value**		
donor (clone and diet)	5.068	4	0.2804		
transplant diet	2.145	1	0.1430		
donor : transplant diet	2.422	4	0.6587		
B. Type II Wald χ^2 tests: only non-toxic transplant diet					
	χ^2	**df**	***P* value**		
donor clone	0.288	2.000	0.8659		
C. pairwise comparisons					
**contrast: donor clones**	**estimate**	**SE**	**df**	***t* ratio**	***P* value**
B7 - B9	0.038	0.112	9	0.340	0.9388
B7 - no microbial inoculum	0.071	0.137	9	0.518	0.8647
B9 - no microbial inoculum	0.033	0.137	9	0.241	0.9687
D. Type II Wald χ^2 tests: only toxic transplant diet					
	χ^2	**df**	***P* value**		
donor clone	7.800	2.000	**0.02024**	*	
E. pairwise comparisons					
**contrast: donor clones**	**estimate**	**SE**	**df**	***t* ratio**	***P* value**
B7 - B9	0.090	0.096	9	0.932	0.6348
B7 - no microbial inoculum	0.327	0.118	9	2.784	0.0506
B9 - no microbial inoculum	0.238	0.118	9	2.023	0.1623
F. Type II Wald χ^2 tests: excluding the treatment without microbial inoculum to separate donor clone and donor diet					
	χ^2	**df**	***P* value**		
donor clone	0.831	1	0.3621		
donor diet	0.047	1	0.8293		
transplant diet	4.121	1	**0.0424**	*	
donor clone : donor diet	0.086	1	0.7696		
donor clone : transplant diet	0.136	1	0.7121		
donor diet : transplant diet	0.003	1	0.9565		
donor clone : donor diet : transplant diet	0.328	1	0.5666		

Table S14: summary statistics for the multivariate mixed effect models to explore the interaction between phenotypic measurements and DNA methylation (arcsine square root transformed). A) The output of the Type II Wald χ^2 tests for the general model. **B)** The output of the Type II Wald χ^2 tests for non-toxic transplant diet. **C)** The trends and pairwise comparisons for the non-toxic transplant diet contrasting the donor clones. **D)** The output of the Type II Wald χ^2 tests for non-toxic transplant diet. **E)** The trends and pairwise comparisons for the non-toxic transplant diet contrasting the donor clones.

Transplant phase					
**Body size and survival percentage**					
A. Type II Wald χ^2 tests: general					
	**χ^2**	**df**	***P* value**		
DNA methylation	17.010	1	**3.718e-05**	***	
donor	183.724	4	**< 2.2e-16**	***	
transplant diet	0.737	1	0.3906		
DNA methylation : donor	14.273	4	**0.0065**	**	
DNA methylation : transplant diet	0.310	1	0.5777		
donor : transplant diet	24.774	4	**5.586e-05**	***	
DNA methylation : donor : transplant diet	15.378	4	**0.0040**	**	
B. Type II Wald χ^2 tests: only non-toxic transplant diet					
	**χ^2**	**df**	***P* value**		
DNA methylation	18.264	1	1.924e-05	***	
microbiome	230.713	4	< 2.2e-16	***	
DNA methylation : microbiome	39.389	4	5.788e-08	***	
C. pairwise comparisons: trends and contrasts (non-toxic transplant diet)					
**trends: donor clones**	**DNA methylation trend**	**SE**	**df**	**lower.CL**	**upper.CL**
B7C	0.901	0.210	55	**0.480**	**1.3200**
B7M	-0.422	2.540	55	-5.512	4.6700
B9C	1.550	0.375	55	**0.799**	**2.3000**
B9M	-1.580	1.400	55	-4.392	1.2300
no microbial inoculum	-2.241	0.555	55	**-3.353**	**-1.1300**
**contrast: donor clones**	**estimate**	**SE**	**df**	***t* ratio**	***P* value**
B7C - B7M	1.3230	2.460	55	0.537	0.6916
B7C - B9C	-0.6490	0.469	55	-1.384	0.3440
B7C - B9M	2.4810	1.500	55	1.656	0.2586
B7C - no microbial inoculum	3.1410	0.653	55	4.810	0.0001
B7M - B9C	-1.9720	2.660	55	-0.740	0.6916
B7M - B9M	1.1580	3.370	55	0.343	0.7325
B7M - no microbial inoculum	1.8180	2.760	55	0.658	0.6916
B9C - B9M	3.1300	1.350	55	2.311	0.0820
B9C - no microbial inoculum	3.7900	0.637	55	5.954	<.0001
B9M - no microbial inoculum	0.6600	1.330	55	0.495	0.6916

D. Type II Wald χ^2 tests: only toxic transplant diet

	χ^2	df	*P* value	
DNA methylation	42.325	1.000	7.729E-11	***
microbiome	163.929	4.000	< 2.2e-16	***
DNA methylation : microbiome	34.518	4.000	5.834E-07	***

E. pairwise comparisons: trends and contrasts (toxic transplant diet)

**trends: donor clones**	**DNA methylation trend**	**SE**	**df**	**lower.CL**	**upper.CL**
B7C	0.792	0.205	54	**0.381**	**1.2000**
B7M	2.353	0.311	54	**1.730**	**2.9800**
B9C	0.202	0.608	54	-1.017	1.4200
B9M	3.779	0.772	54	**2.232**	**5.3300**
no microbial inoculum	0.211	0.568	54	-0.927	1.3500
**contrast: donor clones**	**estimate**	**SE**	**df**	***t* ratio**	***P* value**
B7C - B7M	-1.561	0.321	54	-4.863	**0.0001**
B7C - B9C	0.591	0.588	54	1.004	0.3997
B7C - B9M	-2.987	0.769	54	-3.884	**0.0014**
B7C - no microbial inoculum	0.582	0.663	54	0.877	0.4269
B7M - B9C	2.152	0.654	54	3.287	**0.0036**
B7M - B9M	-1.426	0.754	54	-1.890	0.0915
B7M - no microbial inoculum	2.143	0.707	54	3.032	**0.0062**
B9C - B9M	-3.578	1.010	54	-3.554	**0.0020**
B9C - no microbial inoculum	-0.009	0.937	54	-0.010	0.9924
B9M - no microbial inoculum	3.569	0.999	54	3.572	**0.0020**
